# Homeobox B5 promotes metastasis and poor prognosis in Hepatocellular Carcinoma, via FGFR4 and CXCL1 upregulation

**DOI:** 10.7150/thno.57659

**Published:** 2021-03-31

**Authors:** Qin He, Wenjie Huang, Danfei Liu, Tongyue Zhang, Yijun Wang, Xiaoyu Ji, Meng Xie, Mengyu Sun, Dean Tian, Mei Liu, Limin Xia

**Affiliations:** 1 Department of Gastroenterology, Institute of Liver and Gastrointestinal Diseases, Hubei Key Laboratory of Hepato-Pancreato-Biliary Diseases, Tongji Hospital of Tongji Medical College, Huazhong University of Science and Technology, Wuhan 430030, Hubei Province, China.; 2 Hubei Key Laboratory of Hepato-Pancreato-Biliary Diseases; Hepatic Surgery Center, Tongji Hospital, Tongji Medical College, Huazhong University of Science and Technology; Clinical Medicine Research Center for Hepatic Surgery of Hubei Province; Key Laboratory of Organ Transplantation, Ministry of Education and Ministry of Public Health, Wuhan, Hubei, 430030, China.

**Keywords:** homeobox B5, C-X-C motif chemokine ligand 1, myeloidderived suppressor cell, BLU-554, SB265610

## Abstract

**Background:** Since metastasis remains the main reason for HCC-associated death, a better understanding of molecular mechanism underlying HCC metastasis is urgently needed. Here, we elucidated the role of Homeobox B5 (HOXB5), a member of the HOX transcriptional factor family, in promoting HCC metastasis.

**Method:** The expression of HOXB5 and its functional targets fibroblast growth factor receptor 4 (FGFR4) and C-X-C motif chemokine ligand 1 (CXCL1) were detected by immunohistochemistry. Luciferase reporter and chromatin immunoprecipitation assays were performed to measure the transcriptional regulation of target genes by HOXB5. The effects of FGFR4 and CXCL1 on HOXB5-mediated metastasis were analyzed by an orthotopic metastasis model.

**Results:** Elevated expression of HOXB5 had a positive correlation with poor tumour differentiation, higher TNM stage, and indicated unfavorable prognosis. Overexpression of HOXB5 promoted HCC metastasis through transactivating FGFR4 and CXCL1 expression, whereas knockdown of FGFR4 and CXCL1 decreased HOXB5-enhanced HCC metastasis. Moreover, HOXB5 overexpression in HCC cells promoted myeloid derived suppressor cells (MDSCs) infiltration through CXCL1/CXCR2 axis. Either depletion of MDSCs by anti-Gr1 or blocking CXCL1-CXCR2 axis by CXCR2 inhibitor impaired HOXB5-mediated HCC metastasis. In addition, fibroblast growth factor 19 (FGF19) contributed to the HOXB5 upregulation through PI3K/AKT/HIF1α pathway. Overexpression of FGF15 (an analog of FGF19 in mouse) promoted HCC metastasis, whereas knockdown of HOXB5 significantly inhibited FGF15-enhanced HCC metastasis in immunocompetent mice. HOXB5 expression was positively associated with CXCL1 expression and intratumoral MDSCs accumulation in human HCC tissues. Patients who co-expressed HOXB5/CXCL1 or HOXB5/CD11b exhibited the worst prognosis. Furthermore, the combination of FGFR4 inhibitor BLU-554 and CXCR2 inhibitor SB265610 dramatically decreased HOXB5-mediated HCC metastasis.

**Conclusion:** HOXB5 was a potential prognostic biomarker in HCC patients and targeting this loop may provide a promising treatment strategy for the inhibition of HOXB5-mediated HCC metastasis.

## Introduction

Hepatocellular carcinoma (HCC) is the third-leading cause of cancer-related death and the incidence is increasing 1. Hepatic resection is still the major strategy for early-stage HCC. However, because of the rapid relapse and metastasis, tumor heterogeneity and marginal effect of kinase inhibitors and immunotherapy, treatment of advanced HCC is still challenging 2, 3. Therefore, exploring the underline mechanism of HCC metastasis and finding new therapeutic strategies are still urgently needed. Recently, the combination of atezolizumab and bevacizumab in advanced HCC patients contributes to better overall and progression-free survival outcomes than alone sorafenib treatment 4, which indicates that combination therapy for certain HCC subpopulations is a promising treatment strategy.

Homeobox (HOX) genes with highly conserved homeodomain region contain total 39 transcription factors and are divided into four sub-families named as A, B, C and D in mammals 5. HOX genes play important roles in organ development and regulating apoptosis, receptor signaling, differentiation, motility and angiogenesis 6. Nowadays, numerous studies have reported that deregulation of HOX family genes contributes to tumor initiation, progression and metastasis 6. For example, overexpression of HOXA10 7, HOXA13 8 and HOXB7 9 promotes cancer proliferation, progression and metastasis. However, HOXA5 10, HOXA9 11, HOXB1 and HOXB3 12 function as tumor suppressor genes (TSGs) to suppress tumor growth and metastasis. Moreover, HOXB13 is identified as either a tumor suppressor or oncogene in different tumor types 13-15. HOXB subfamily locates in chromosome 17 and consists of 10 family members, named HOXB1-10 16. HOXB5, one member of HOXB subfamily, is important for T lymphocytes generation 17, vascular remodeling 18, neural crest development 19, and angiogenesis 20. Meanwhile, HOXB5 functions as an oncogene in several cancers like breast cancer 21, retinoblastoma 22, and small cell lung cancer 23. Previously, we analyzed the mRNA expression levels of 39 HOX genes in 10 normal liver tissues and 30 pairs of HCC tissues and adjacent nontumor tissues. Apart from HOXC10, we also found that the mRNA levels of HOXB5 were significantly upregulated in HCC tissues, and knockdown of HOXB5 inhibited the migration and invasion abilities of HCC cell line HCCLM3 24. Another study reported that HOXB5 is upregulated in HCC tissues and overexpression of HOXB5 promoted HCC cell proliferation and inhibited cell apoptosis 25. These studies suggested that HOXB5 may play a role in HCC progression. However, whether HOXB5 is involved in HCC metastasis remains unknown. Its underlying molecular mechanism needs further investigation.

Fibroblast growth factor receptor 4 (FGFR4), a special receptor of fibroblast growth factor 19 (FGF19), features in inducing myogenesis, regulating metabolism of lipid, bile acid and glucose, and coordinating cell proliferation and differentiation 26. The gene copy amplification of FGF19 and the elevated expression of FGFR4 are validated as oncogenic drivers 27, 28 and indicate poor prognosis in HCC 29. FGF19-FGFR4 signaling activates several signaling pathways including extracellular regulated protein kinase (ERK), Jun N-terminal kinase (JNK), phosphoinositide 3-kinase (PI3K), protein kinase C (PKC), mammalian target of rapamycin (mTOR), and signal transducer and activator of transcription 3 (STAT3) to promote HCC carcinogenesis, progression and metastasis^29^. Inhibition of FGF19-FGFR4 signaling by FGFR4 specific inhibitor BLU-554 suppresses HCC progression 30. All these evidences demonstrate that FGF19-FGFR4 signaling pathway plays a critical role in promoting HCC progression and metastasis. Nevertheless, the roles of dysfunctional FGF19-FGFR4 pathway on HCC metastasis needs more study.

Myeloid-derived suppressor cells (MDSCs) are initially found to protect the host from extensive tissue damage. Tumors hijack and amplify this role to induce immunosuppressive effect by suppressing CD8^+^ T cell proliferation and activation 31. In HCC tumor microenvironment, several cytokines and chemokines such as CXCL1, CSF1, CCL2, CCL9, IL-6, and IL-18 etc., which were secreted from either HCC cells or immune cells, promotes the recruitment and infiltration of MDSCs from the bone marrow into HCC tumor site 32-34. MDSCs boost HCC progression through inducing immune suppression 35, impairing antitumor efficacy of cytokine-induced killer (CIKs) 36, and fostering tumour-supporting inflammation 37. Several studies have demonstrated that MDSCs infiltration in HCC predicts poor prognosis 2, 32, 38. All these evidences prove that MDSCs accumulation accelerates HCC progression and metastasis. In additional, several methods in targeting MDSCs has been used to impair HCC progression such as: suppressing MDSCs suppressor function 36, decreasing the recruit of MDSCs through targeting special receptors in MDSCs via antagonist or monoclonal antibody and inhibiting cytokines 33, inducing rapid apoptosis of MDSCs 35, and abrogating monocyte differentiation 2. However, tumour-intrinsic oncogenic signaling and the exact mechanism in promoting MDSC accumulation in HCC are still unknown. More importantly, the combined treatment value of targeting MDSCs in HCC is unknown.

In this study, we found that FGF19 upregulated HOXB5 expression, and overexpression of HOXB5 promoted HCC metastasis through upregulating FGFR4 and CXCL1. The combination of FGFR4 inhibitor BLU-554 and CXCR2 inhibitor SB265610 dramatically suppressed HOXB5-mediated HCC metastasis.

## Methods

### Establishment of Orthotopic HCC Models

BALB/C nude mice (male, 5 weeks old) and C57BL/6 mice (male, 5 weeks old) were housed under standard conditions and cared for according to the institutional guidelines for animal care. The animal experiments were authorized by the Ethics Committee on the Tongji Hospital of Tongji Medical College, Huazhong University of Science and Technology. Total 2×10^6^ PLC/PRF/5, MHCC97H or Hepa1-6 cells transfected with the indicated lentivirus were prepared for orthotopic inoculation and resuspended these cells in 50 μL PBS/matrigel mixture. Mice (ten per group) were anesthetized and orthotopically injected these indicated cells to the left lobe of liver by using a microsyringe in the epigastrium with an 8 mm incision. 9 weeks after the treatment or when the mice were very weak, the livers and lungs were collected for further evaluation at the end of the experiment.

### *In vivo* treatment studies

For the MDSC depletion, the mice treated with Gr-1 monoclonal antibody or IgG through intraperitoneal injection twice a week (2 mg/kg) and SB265610 (2 mg/kg body weight) or PBS was injected i.p. every day for inhibiting the CXCR2 receptor. For combined treatment, mice were injected intraperitoneally with SB265610 (2 mg/kg body weight) or 10 mg/kg BLU-554 orally daily.

### Immunofluorescence (IF)

Formalin-fixed paraffin-embedded sections (4 μm) were baked, deparaffinized, rehydrated, followed by antigen retrieval and permeabilized. After that the tissues were blocked with 10% goat or donkey serum for 30 minutes and incubated with primary antibodies at 4°C overnight. After washing, appropriate secondary antibodies were used. The diamidine phenylindole (DAPI) was used to stain cell nucleus for ten minutes. Florescence was visualized under an Olympus fluorescence microscope.

### Preparation of Single Cell Suspensions

Prior to flow cytometry analysis, single cell suspensions should be prepared. The method was used as described in the research paper 32. Briefly, after the anesthetization of mice, Hank's buffer without calcium was first injected into the liver through the portal vein. After that, the Hank's buffer with calcium, magnesium and collagenase IV (0.2 mg/mL, Sigma-Aldrich, C5138) was injected into the liver. After separation of the liver and tumor, the tissues were made into small pieces about 1mm^3^. Mouse tumor dissociation buffer (Miltenyi, 130-096-730) was used to prepare the single cell by using the gentleMACS dissociator (Miltenyi Biotech) followed by filtration through a 70μm cell mesh, lysing erythrocyte, centrifuging and resuspending in Hank's buffer.

### Flow cytometry

After the anesthetization of mice, tumors were collected to prepare the single cell suspensions according to the procedure described above. Fc block was added to the cells at room temperature for 10 minutes and then incubated with primary antibodies or isotype antibodies at 4°C for 45 minutes. A FACS LSRFortessa and FlowJo software (BD Biosciences) were used to acquire and analyze the data respectively.

### Chromatin immunoprecipitation Assay (ChIP)

Cells were immersed in 1% formaldehyde for 10 minutes at 37 °C to stimulate cross-linking. Then, glycine was used to quench the formaldehyde after cross-linking to stop formaldehyde fixation. After washing with PBS, the cells were resuspended in lysis buffer (1 mM PMSF, 1% SDS, 10 mM EDTA and 50 mM Tris (pH 8.1) - total volume 300 μl). Sonication was then performed to produce fragmented DNA. A slurry of protein G-Sepharose and herring sperm DNA (Sigma-Aldrich) was used to clear the supernatant. The recovered supernatant was then subjected to a 2-hour incubation period with specific antibodies or an isotype control IgG in the presence of protein G-Sepharose beads and herring sperm DNA, followed by antibody denaturation with 1% SDS in lysis buffer. Precipitated DNA was extracted from the beads by immersing them in a 1.1 M NaHCO3 solution and 1% SDS solution at 65 °C for 6 hours. Immunoprecipitated DNA was retrieved from the beads by immersion in 1% SDS and a 1.1 M NaHCO3 solution at 65 °C for 6 hours. The DNA was then purified using a PCR Purification Kit (QIAGEN, USA). The primers were shown in Supplementary Table S9.

For ChIP assays of tissues, cells were first separated from six pairs of fresh frozen HCC tissues and normal liver tissues collected after surgical resection. In detail, surgically extracted tumor tissues were first washed by 1× cold, PBS, 5 min, for three times and added to medium supplemented with antibiotic and antifungal agents. Use a clean razor blade to cut a pie of tissue (around 5 mm^3^) into small piece (typical 1 mm^3^ or smaller). Then, digestion the tissues with DNase I (20 mg/mL; Sigma-Aldrich) and collagenase (1.5 mg/mL; Sigma-Aldrich) and placed on table concentrator, 37°C, for 1 h. At the end of the hour, we filtered the dissociated cells through 100-μm-pore filters rinsed with fresh media. The 1×red cell lysis was added to the tissues and incubated for 5 minutes to lysis the red blood cell, followed by another rinse. The dissociated cells were crosslinked using 1% formaldehyde for 10 minutes at 37°C. After cell lysis, the DNA was fragmented by sonication. ChIP grade antibody or IgG (negative control) was used to immunoprecipitated the fragment DNA. Then, qRT-PCR was used to amplify the corresponding binding site on the promoters.

### Statistical analysis

All values were recorded as the mean ± standard deviation (s.d.). All experiments were repeated three or more independent biological replicates. Statistical significance between the means of two groups was determined using Student's t tests (normal distribution), Mann-Whitney U tests (abnormal distribution) or Wilcoxon signed rank test (matched pairs). The statistics of the means of multiple groups were performed using one way ANOVA or two-way ANOVA. Immunohistochemical score was analyzed by chi-squared test. The cumulative recurrence and survival curves were shown by the Kaplan-Meier method and the statistical significance were determined by log-rank test. Multivariate analysis was performed by Cox regression analysis. Correlations were performed by using a Pearson correlation test. Statistical analysis was justified as appropriate among all figures. P values < 0.05 were considered to be statistically significant. Statistical values were calculated with SPSS software (Version 20.0) or GraphPad Prism 8.0 software.

More details about the methods and materials are available in the Supplementary Materials.

## Results

### HOXB5 upregulation boosts HCC metastasis and indicates unfavorable prognosis in human HCC

**Figure 1:**
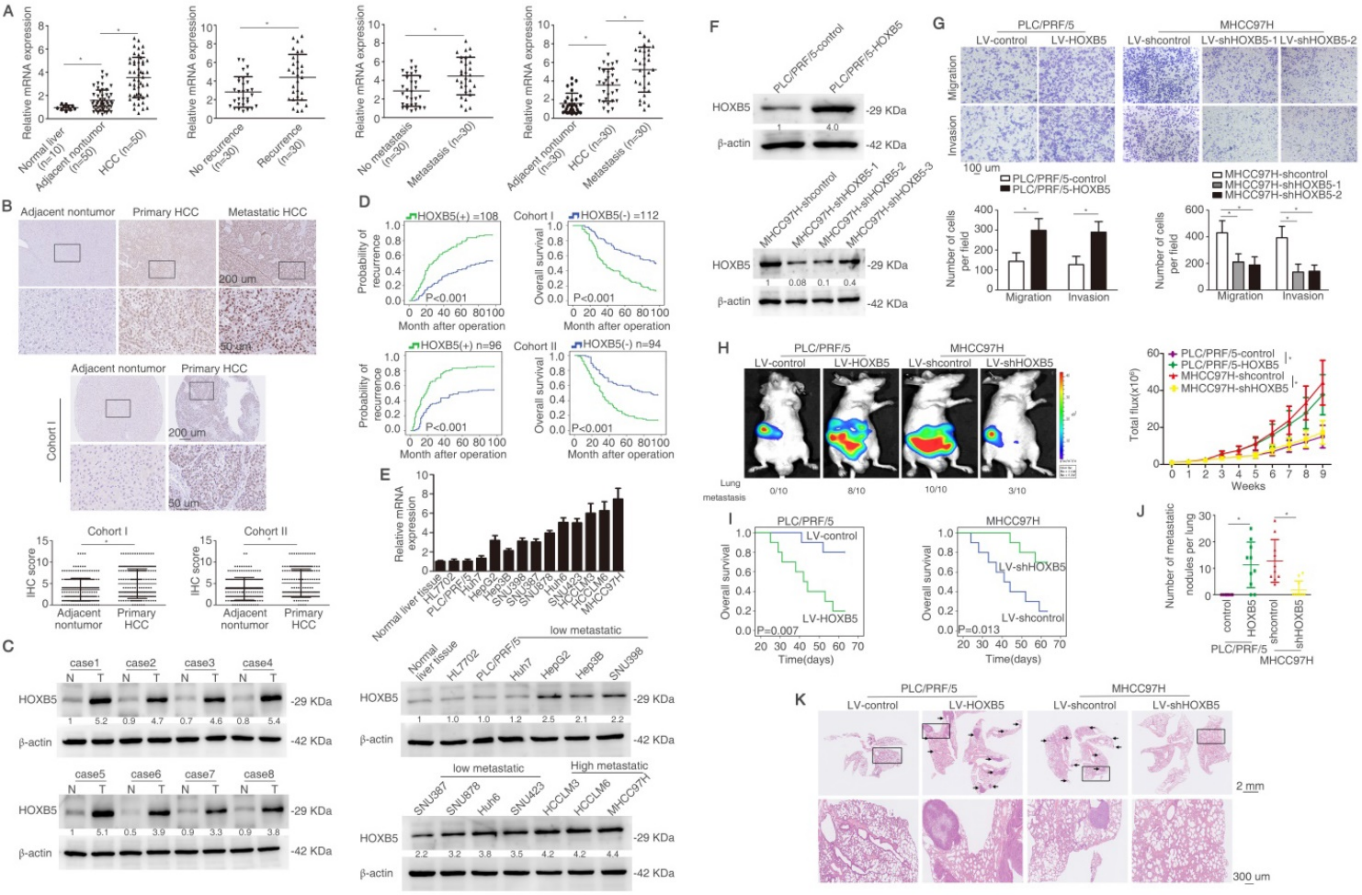
** HOXB5 upregulation boosts HCC metastasis and indicates unfavorable prognosis in human HCC.** (A) RT-qPCR was used to detect the HOXB5 mRNA expression in 10 normal liver tissues and 50 paired adjacent nontumorous and HCC tissues, in with or without recurrent HCC patient samples (n=30), in 30 paired metastatic samples, and in 30 paired HCC tissues and metastatic HCC tissues. (B) Representative IHC images of HOXB5 expression were shown in adjacent nontumorous tissues, HCC tissues and metastatic HCC tissues (upper). Representative IHC images was shown from cohort I (middle). IHC scores of HOXB5 in two HCC cohorts were shown (lower). Data was analyzed by chi-squared test. (C) Western blotting showed the protein expression of HOXB5 in paired HCC tissues. (D) Kaplan-Meier was applied to analyze the association of HOXB5 expression and recurrence rate or overall survival time in two HCC cohorts. (E) Relative mRNA (upper) and protein expression (lower) of HOXB5 were shown in HCC cell lines and normal liver tissues. (F) Western blotting was used to detect HOXB5 expression in indicated cells. (G) Transwell showed the capability of migration and invasion in HCC cells after the changes of HOXB5 expression. (H-K) *In vivo* assays showed that HOXB5 knockdown can inhibit HCC metastasis. (H) Bioluminescent images, growth rate and lung metastasis rate were shown. (I) Survival curve was shown. (J) metastatic lung nodules were counted. (K) HE staining was applied to exhibit metastatic lung nodules. * P < 0.05.

In order to detect the expression of HOXB5 in HCC, we detected its mRNA expression in an HCC cohort with 50 paired tissues. We found that the mRNA level of *HOXB5* was higher in HCC tissues than that in normal liver tissues and adjacent non-tumorous tissues (Figure 1A left). The *HOXB5* mRNA levels were upregulated in HCC tissues with recurrent or metastatic patients compared to these in patients without recurrence or metastasis (Figure 1A middle). Furthermore, mRNA expression of *HOXB5* was higher in metastatic HCC tissues than that in primary HCC and adjacent nontumor tissues (Figure 1A right). The immunohistochemical (IHC) images showed higher HOXB5 expression in metastatic HCC tissues than in primary HCC tissues (Figure 1B upper).

We then detected the protein expression and analyzed the clinical importance of HOXB5 in HCC cohorts 39. IHC staining was used to detect the expression of HOXB5 in cohort I with 220 patient samples. We first ascertain specificity of HOXB5 antibody for IHC experiment (Figure S1A-B). HOXB5 expression was higher in HCC tissues compared to adjacent non-tumorous tissues (Figure 1B, middle). Similarly, western blotting analysis showed that HOXB5 expression was upregulated in HCC tissues than in paired adjacent nontumorous tissues (Figure 1C). Positive HOXB5 expression in HCC patients contributed to higher recurrence rate and shorter overall survival time compared to patients with negative HOXB5 expression (Figure 1D). The positive HOXB5 expression was positively correlated with loss of tumor encapsulation, microvascular invasion, poorer differentiation and higher tumor-nodule-metastasis (TNM) stage (Table S1). According to the result of multivariate analysis, we found that HOXB5 was a valuable factor for predicting recurrence rate and survival time in HCC patients (Table S2). We next applied an independent HCC cohort (Cohort II, n=190) to further evidence HOXB5 expression and clinical significance. Similarly, positive expression of HOXB5 indicated poor prognosis (Figure 1D), and HCC tissues with positive HOXB5 expression was positively correlated with loss of tumor encapsulation, microvascular invasion, poor differentiation and higher TNM stage (Table S1). Multivariate analysis manifested that HOXB5 was a significant biomarker for predicting postoperative recurrence rate and overall survival time (Table S2). All these works suggested that HOXB5 was a prognostic predictor in HCC patients.

RT-qPCR and western blotting were used to detect the HOXB5 expression in human HCC cell lines with different metastatic potency 29, 40-42. HOXB5 expression was higher in metastatic HCC cells lines than in HCC cells with low metastatic ability (Figure 1E). We found that HOXB5 expression was relatively low expression in PLC/PRF/5 cell and relatively high expression in MHCC97H cells. Therefore, we chose PLC/PRF/5 cells to upregulate HOXB5 expression and MHCC97H cells to downregulate HOXB5 expression. PLC/PRF/5 and MHCC97H cells were selected to establish stable cell lines, PLC/PRF/5-HOXB5, MHCC97H-shHOXB5 through lentivirus infection (Figure 1F). Our previous work has demonstrated that HOXC10, belonging to the same family with HOXB5, can promote HCC metastasis. We first detected whether HOXB5 changes had any influence on HOXC10 expression. The results demonstrated that HOXB5 had no had no significant changes on HOXC10 expression (Figure S1C). The transwell assay indicated that the upregulation of HOXB5 promoted the migratory and invasive ability of PLC/PRF/5 cells, while HOXB5 downregulation decreased the migratory and invasive ability of MHCC97H cells (Figure 1G). We then performed the *in vivo* metastatic assay. Bioluminescent images showed that upregulation of HOXB5 promoted the growth of liver tumors established by PLC/PRF/5 cells and HOXB5 downregulation decreased the growth of liver tumors established by MHCC97H cells (Figure 1H). Upregulation of HOXB5 lowered the survival time of the nude mice and knockdown of HOXB5 expression extended the survival time of mice in MHCC97H group (Figure 1I). Elevated metastatic lung nodules were shown in PLC/PRF/5-HOXB5 group and knockdown of HOXB5 expression largely impaired metastatic lung nodules (Figure 1J). Figure 1K showed HOXB5 overexpression can promote lung metastasis of PLC/PRF/5 cells and HOXB5 downregulation can decrease lung metastasis of MHCCP7H cells shown by HE staining. These studies indicated that HOXB5 promoted HCC metastasis.

After metastatic cell entered the circulatory blood system, they needed to initiate and maintain growth for a macroscopic tumor to form 43, 44. Therefore, we also detected the roles of HOXB5 in HCC progression. We first detected the effect of HOXB5 on HCC proliferation *in vitro*. Cell Counting Kit-8 (CCK8) and colony formation assays demonstrated that HOXB5 upregulation can increase HCC cell proliferation of PLC/PRF/5 cells, whereas HOXB5 knockdown impaired HCC cell proliferation of MHCC97H cells (Figure S2A and B). We then performed the *in vivo* analysis. *In vivo* tumorigenicity assays illustrated that HOXB5 upregulation increased tumor growth of PLC/PRF/5 cells, whereas HOXB5 knockdown impaired tumor growth of MHCC97H cells (Figure S2C-D). IHC staining for Ki67 was used to confirm that HOXB5 can promote HCC proliferation* in vivo* (Figure S2E). These studies orchestrated that HOXB5 promoted HCC cell progression.

### HOXB5 promotes HCC metastasis through upregulating FGFR4 expression in immunodeficient mice

**Figure 2:**
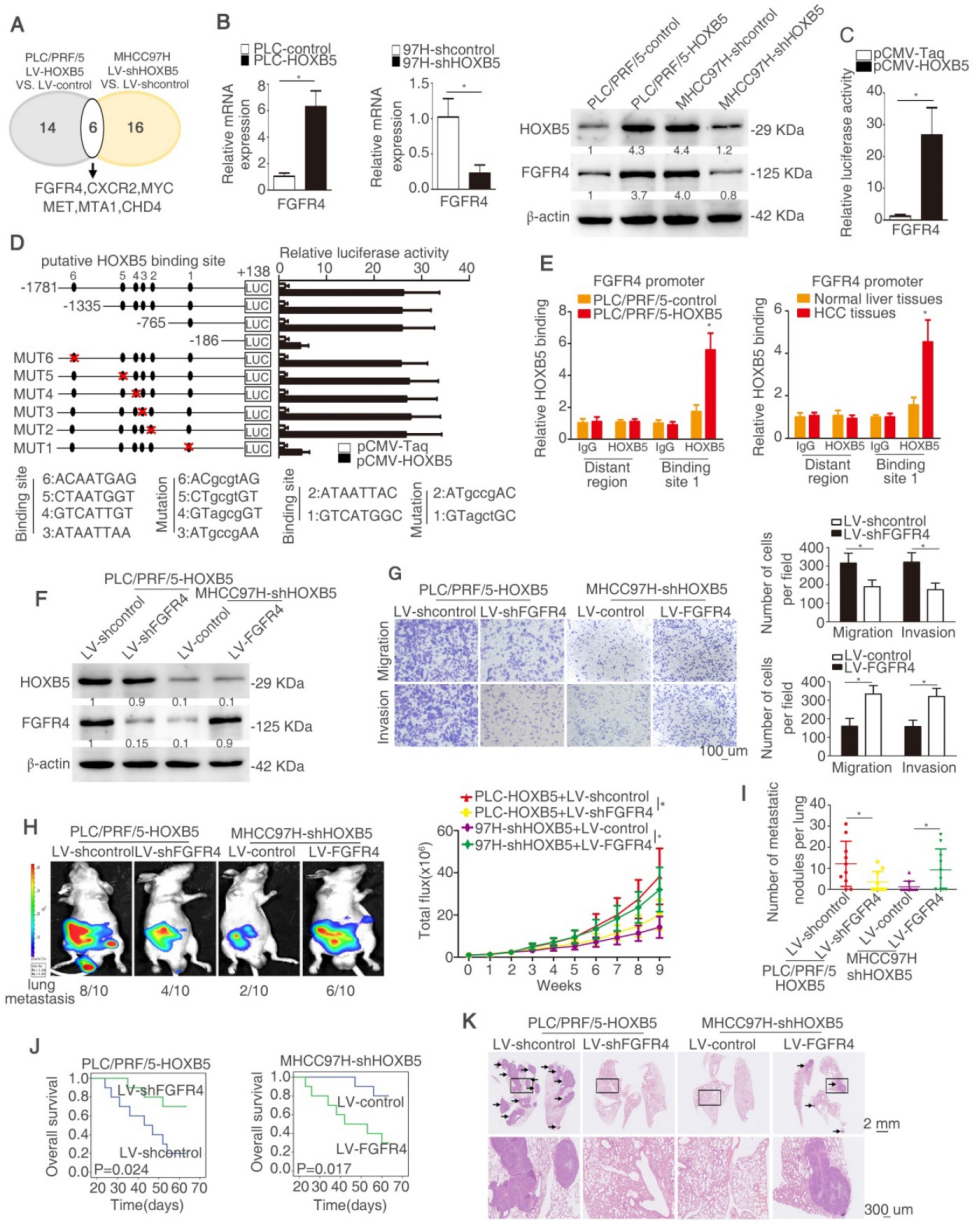
** HOXB5 promotes HCC metastasis through upregulating FGFR4 expression in immunodeficient mice.** (A) The diagram showed the genes regulated by HOXB5 changes. (B) RT-qPCR and western blotting were used to show FGFR4 level in the indicated cells. (C) Cells were co-transfected with luciferase construct containing FGFR4 promoter and pCMV-HOXB5 and Luciferase reporter activity was analyzed. (D) Luciferase activity was measured after the transfection of truncated and mutated FGFR4 promote and pCMV-HOXB5. (E) ChIP assays revealed the interacting of HOXB5 and FGFR4 promoter in HCC cells and in HCC specimens. (F) Western blotting showed HOXB5 and FGFR4 expression in PLC/PRF/5-HOXB5 transfected with LV-shcontrol or LV-shFGFR4 and in MHCC97H-shHOXB5 transfected with LV-control or LV-FGFR4. (G) Transwell showed the capability of migration and invasion in PLC/PRF/5-HOXB5 cells with downregulation of FGFR4 and in MHCC97H-shHOXB5 cell with FGFR4 overexpression. (H-K) *In vivo* assays exhibited that HOXB5 promoted HCC metastasis through upregulating FGFR4. (H) Bioluminescent images and grow rate were shown. (I) Lung metastatic nodules were counted. (J) Overall survival time of mice in different groups was shown. (K) Lung metastatic nodules in different groups were shown. * P < 0.05.

In order to explore the mechanism underlying HOXB5-mediated HCC metastasis, a human tumor metastasis PCR array was applied to test the changes in mRNA profile induced by HOXB5 change. To designate differentially expressed genes in tumor metastasis PCR arrays, we used twofold as a cut-off. Fourteen out of 84 genes were upregulated in PLC/PRF/5-HOXB5 compared with PLC/PRF/5-control cells. Sixteen out of 84 genes were downregulated in MHCC97H-shHOXB5 compared with MHCC97H-shcontrol cells. Among the overlapped six genes, FGFR4 attracted our attention, which were significantly upregulated in HOXB5 overexpressing cell PLC/PRF/5-HOXB5 and were downregulated in HOXB5 knockdown cell MHCC97H-shHOXB5 (Figure 2A, Table S3-4). Because of momentous function of FGFR4 in HCC progression, we then determined whether HOXB5-induced HCC metastasis was dependent on FGFR4. Overexpression of HOXB5 upregulated FGFR4 expression, however HOXB5 knockdown impaired FGFR4 expression (Figure 2B). Moreover, HOXB5 changes had no significant influence on FGFR1, FGFR2 and FGFR3 (Figure S1D). Luciferase activity of *FGFR4* promoter was promoted after the upregulation of HOXB5 in PLC/PRF/5 cells compared to the control group (Figure 2C).

In order to identify how HOXB5 regulates FGFR4 expression, the *FGFR4* promoter was detected and six putative HOXB5 binding motifs were found in the *FGFR4* promoter. We generated a series of the truncation or mutation of *FGFR4* promoter sequence. We found that the luciferase reporter activity was decreased under the deletion of sequence between -765bp to -186bp, which suggested that this region was crucial for HOXB5-induced FGFR4 expression. In order to further explore how this region regulates the effect, we found one HOXB5 binding site located in this region. The mutation of HOXB5 showed that the binding site 1 in the *FGFR4* promoter contributed to the increased luciferase activity induced by HOXB5 (Figure 2D). Moreover, the results of chromatin immunoprecipitation (ChIP) demonstrated that HOXB5 interacted with *FGFR4* promoter directly in PLC/PRF/5-HOXB5 cells and human HCC samples (Figure 2E). All these findings demonstrated that HOXB5 promoted *FGFR4* expression through direct binding with its promoter.

To explore whether FGFR4 was involved in HOXB5-induced HCC metastasis, we knocked down FGFR4 expression in HOXB5-overexpressing cell PLC/PRF/5-HOXB5 and ectopically upregulated FGFR4 expression in MHCC97H-shHOXB5 cells (Figure 2F). Knockdown of FGFR4 significantly decreased migratory and invasive abilities of PLC/PRF/5-HOXB5 cells, whereas overexpression of FGFR4 rescued the migratory and invasive capabilities of MHCC97H-shHOXB5 cells (Figure 2G, Figure S1E). *In vivo* metastatic analysis showed that decreased expression of FGFR4 lowered lung metastasis rate and decreased metastatic lung nodules and prolonged the overall survival time of nude mice in PLC/PRF/5-HOXB5 group (Figure 2H-K). In contrast, overexpression of FGFR4 reversed the impaired lung metastasis and reduced metastatic lung nodules in MHCC97H-shHOXB5 group and decreased the survival time of these mice in this group (Figure 2H-K). These results illustrated that HOXB5 promoted HCC metastasis by upregulating FGFR4 expression in immunodeficient mice.

The research works have demonstrated that FGFR4 can promote HNSCC metastasis via EGFR/Akt/Wnt/β-catenin signaling axis 45 and induce EMT in breast Cancer Cells 21. Therefore, we intended to detect whether HOXB5 can active β-catenin pathway and induce EMT. As shown in the figure S1F, HOXB5 upregulation activated β-catenin and induced EMT. Knockdown of FGFR4 inhibited β-catenin activation and impaired EMT. According to these results, decreased β-catenin signaling and EMT may contribute to HOXB5-indeced HCC metastasis by upregulating FGFR4.

### Knockdown of FGFR4 partially inhibits HOXB5-promoted HCC metastasis in immunocompetent mice

In order to detect whether HOXB5-FGFR4 axis strengthened HCC metastasis in immunocompetent mice, we used C57BL/6 mice to establish a model by injecting Hepa1-6 cells into the livers. Hepa1-6 cells have low endogenous HOXB5 and FGFR4 expression (Figure S1G). We established stable cell line Hepa1-6-HOXB5 and knocked down FGFR4 expression in Hepa1-6-HOXB5 cells (Figure S3A). Overexpression of HOXB5 facilitated HCC metastasis in immunocompetent mice. However, knockdown of FGFR4 just partly inhibited the HOXB5-induced HCC metastasis (Figure S3B-C) and had no significant influence on survival time of mice (P=0.047, Figure S3D). FGFR4 downregulation partly decreased the metastatic nodules in the lung shown in Figure S3E and Figure S3F. We then detected the CD8^+^ T cells in the orthotopic tumors. We found that overexpression of HOXB5 in Hepa1-6 cells resulted in a decrease of CD8^+^T cells in orthotopic tumors and knockdown of FGFR4 in Hepa1-6-HOXB5 cells increased the number of CD8^+^T cells. There is no statistical significance between Hepa1-6-control group and Hepa1-6-HOXB5+LV-shFGFR4 group (Figure S3G). These results demonstrated that HOXB5 can influence the immune system and knockdown of FGFR4 just partially reversed the HOXB5-induced immune dysfunction. These results suggested that immunosuppression may participate in HOXB5-induced HCC metastasis. These results suggested that immunosuppression may participate in HOXB5-induced HCC metastasis.

### HOXB5 promotes HCC metastasis through transactivating CXCL1 expression

**Figure 3:**
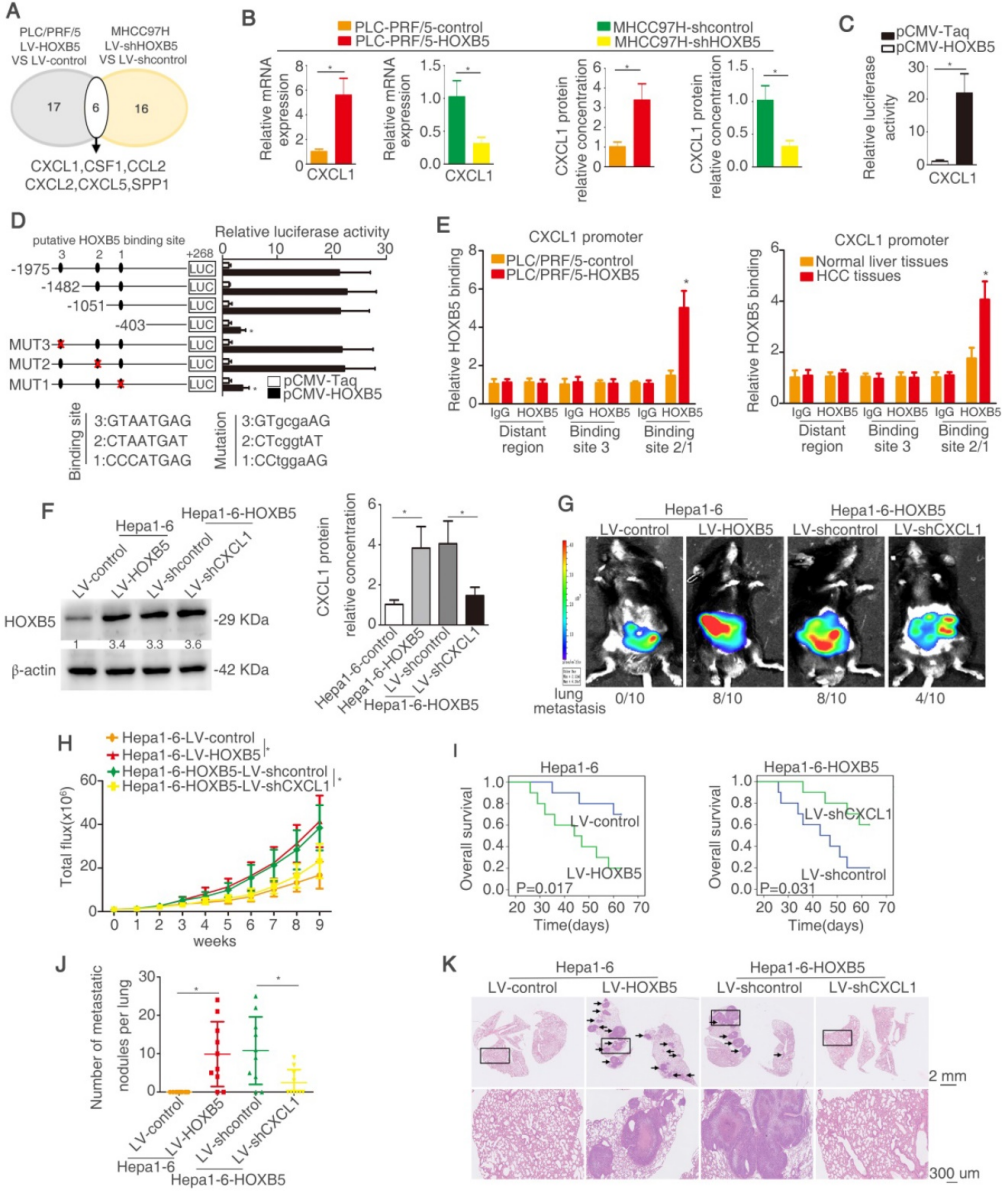
** HOXB5 promotes HCC metastasis through transactivating CXCL1 expression.** (A) The diagram showed the genes regulated by HOXB5 changes. (B) RT-qPCR and ELISA were used to detect the CXCL1 expression. (C) Luciferase reporter assay was performed after co-transfection of CXCL1 promoter luciferase construct and pCMV-HOXB5. (D) Cells were first co-transfection of pCMV-HOXB5 and truncated and mutated CXCL1 promoter and relative luciferase activity was measured. (E) ChIP assays revealed the binding of HOXB5 in CXCL1 promoter in HCC cell lines and HCC specimens. (F) Western blotting and ELISA analyzed protein expression of CXCL1 in the indicated HCC cells. (G-K) *In vivo* assays showed that HOXB5 promoted HCC metastasis through upregulating CXCL1. (G-H) The C57BL/6 mice were implanted with the indicated cells in the liver. Bioluminescent images and growth rate were shown. (I) Overall survival time of C56BL/6 mice in different groups was shown. (J) Lung metastatic nodules were counted. (K) Lung metastatic nodules in different groups. * P < 0.05.

Myeloid cells including TAMs and MDSCs in tumor microenvironment are proved to play important roles in promoting tumor progression through inducing immunosuppression 35, 36, 46. Cytokines and chemokines have been evidenced to enhance HCC metastasis through recruiting these myeloid cells 47. To investigate whether cytokines or chemokines were involved in HOXB5-mediated HCC metastasis, a human Cytokines & Chemokines PCR Array was applied to compare cytokines and chemokines expression in PLC/PRF/5-HOXB5 cell and PLC/PRF/5-control cell or MHCC97H-shHOXB5 cell and MHCC97H-shcontrol cell. Using twofold as a cut-off, overexpression of HOXB5 upregulated 17 cytokines and chemokines expression in PLC/PRF/5 cells, whereas knockdown of HOXB5 decreased 16 cytokines and chemokines expression in MHCC97H cells (Figure 3A, Table S3-4). Among the overlapped 6 genes, CXCL1 attracted our attention. CXCL1 interacts with its receptor CXCR2 to recruit MDSCs 48, 49. Previous studies indicated that tumor cell-derived CXCL1 promotes the recruitment and infiltration of MDSCs to the tumor site and facilitates HCC metastasis 32. We hypothesized whether CXCL1-induced MDSCs infiltration is involved in HOXB5-promoted HCC metastasis.

PLC/PRF/5 cells have low endogenous CXCL1 expression, whereas MHCC97H cells have relatively high endogenous CXCL1 expression (Figure S1H). The RT-qPCR and ELISA assays showed that overexpression of HOXB5 promoted CXCL1 expression and secretion from PLC/PRF/5 cells. However, knockdown of HOXB5 decreased CXCL1 expression and secretion from MHCC97H cells (Figure 3B). Luciferase reporter assay illustrated that upregulation of HOXB5 increased the *CXCL1* promoter activity in PLC/PRF/5 cells (Figure 3C). In order to testify how HOXB5 regulated CXCL1 expression, the promoter of *CXCL1* was detected and three potential HOXB5 binding motifs were found in the *CXCL1* promoter and then the truncated or mutated *CXCL1* promoter sequence were produced. We found that luciferase reporter activity was decreased when the sequence between -1051bp to -403bp was removed. This result indicated that this region was important for HOXB5-induced CXCL1 upregulation. In this region, one HOXB5 binding site was found. We then used the sequence with mutated binding site. The results showed that binding site one mutation largely decreased the luciferase activity. this result illustrated that binding site 1 was essential for HOXB5-induced CXCL1 expression (Figure 3D). Moreover, ChIP showed direct interaction between HOXB5 and *CXCL1* promoter in PLC/PRF/5-HOXB5 cells and HCC tissues (Figure 3E). These findings demonstrated that HOXB5 promoted CXCL1 expression and secretion from HCC cells through directly binding to its promoter.

Hepa1-6 with a relatively low expression compared to H22 (Figure S1I). To investigate whether CXCL1 participates in HOXB5-mediated HCC metastasis, we then used the knocked down CXCL1 expression with lentivirus transduction in Hepa1-6-HOXB5 cells (Figure 3F, Figure S1J). *In vivo* metastatic assay showed that knockdown of CXCL1 reduced lung metastasis rate and metastatic lung nodules while extending the survival time of mice in Hepa1-6-HOXB5 group (Figure 3G-K). These studies suggested that CXCL1 was involved in HOXB5-mediated HCC metastasis.

### HOXB5 promotes HCC metastasis through CXCL1/CXCR2 pathway-induced MDSCs infiltration

**Figure 4:**
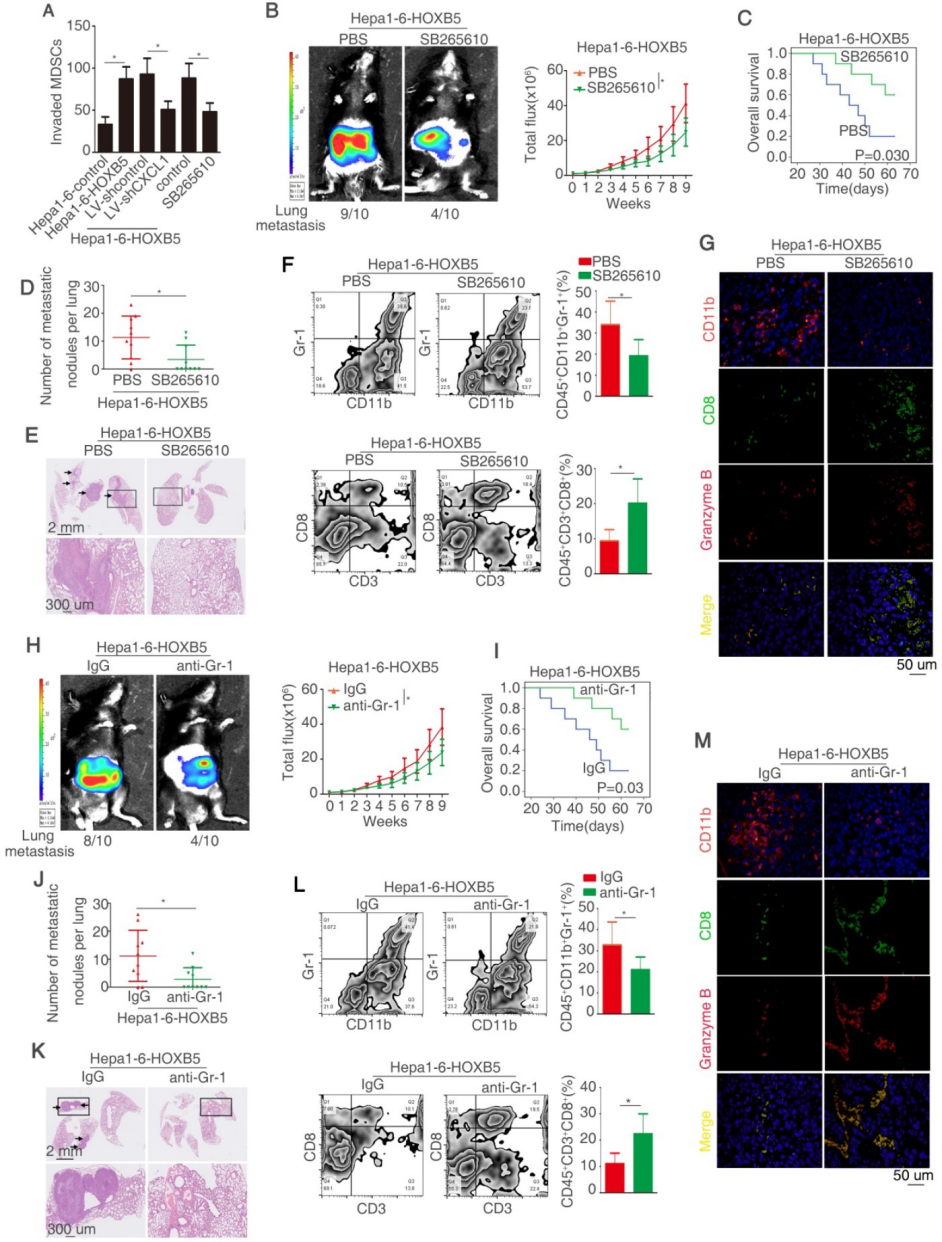
** HOXB5 promotes HCC metastasis through CXCL1/CXCR2 pathway-induced MDSCs infiltration.** (A) MSDCs treated with conditioned media from Hepa1-6-HOXB5 cells with or without LV-shCXCL1 and SB265610 and migratory ability of MDSCs was shown. Bars represented the means ± SD of 3 independent experiments. (B-E) *In vivo* assays showed that CXCR2 inhibitor blocked the HOXB5-promoted HCC metastasis. (B) The indicated cells were implanted into the liver of C57BL/6 mice and bioluminescent images were shown. (C) Overall survival time of C56BL/6 mice was shown. (D) The number of lung metastatic nodules was counted. (E) HE staining showed metastatic nodules in the mice lung in different groups. (F) MDSCs and CD8^+^T cell were analyzed by flow cytometry. (G) IF showed the infiltration of MDSCs and CD8^+^T cell in different groups. (H-K) *In vivo* assay shown that anti-Gr-1 blocked the HOXB5-induced HCC metastasis. (H) The liver of C57BL/6 mice was implanted with cells and bioluminescent images in were shown. (I) Overall survival time of C56BL/6 mice in different groups was shown. (J) Lung metastatic nodules were counted. (K) HE staining shown metastatic nodules in the lung in different groups. (L) MDSCs and CD8^+^T cell were analyzed by flow cytometry. (M) The infiltration of MDSCs and CD8^+^T cell in different groups was shown by IF.

CXCL1 majors in attracting MDSCs to the tumor site by interacting with its special receptor CXCR2 32. Under this context, we explored whether HOXB5-induced CXCL1 secretion from HCC cells promotes the recruitment and infiltration of MDSCs. The migratory ability of MDSCs was increased after treatment with conditional medium from Hepa1-6-HOXB5 cells compared with those treated with conditional medium from Hepa1-6-control cells. Either CXCL1 knockdown or treatment with CXCR2 inhibitor SB265610 significantly impaired the migration ability of MDSCs promoted by the conditional medium from Hepa1-6-HOXB5 cells (Figure 4A). The treatment of recombination CXCL1 promoted the MDSCs migration (Figure S1K). To investigate whether MDSCs infiltration were involved in HOXB5-mediated HCC metastasis, CXCR2 inhibitor SB265610 was used to decrease the chemotaxis of MDSCs *in vivo*. The results showed that SB265610 treatment reduced the lung metastasis rate and metastatic lung nodules while increased the survival time of mice in Hepa1-6-HOXB5 group (Figure 4B-E). Flow cytometry showed that SB265610 treatment resulted in a decrease of the number of MDSCs that was marked with CD45^+^CD11b^+^Gr-1^+^ and an increase of the number of CD8^+^T cells marked with CD45^+^CD3^+^CD8^+^ in the orthotopic tumors (Figure 4F). Granzyme B expression represents T cell activity. Immunofluorescence (IF) staining showed that SB265610 treatment impaired the infiltration of MDSCs and improved the amount and activity of CD8^+^T cells (Figure 4G). We next used the anti-Gr-1-antibody to deplete the MDSCs. *In vivo* metastatic assay showed that depletion of MDSCs by anti-Gr-1 reduced lung metastasis rate and metastatic lung nodules while increased overall survival time of mice in Hepa1-6-HOXB5 group (Figure 4H-K). Flow cytometry and IF staining showed that depletion of MDSCs lowered the accumulation of MDSCs and increased the amount and activity of CD8^+^T cells (Figure 4L-M). These results demonstrated that CXCL1-CXCR2 axis induced MDSCs infiltration is essential for HOXB5-mediated HCC metastasis.

### HOXB5 expression is positively correlated with CXCL1 expression and intratumoral MDSCs infiltration in human HCC tissues

**Figure 5:**
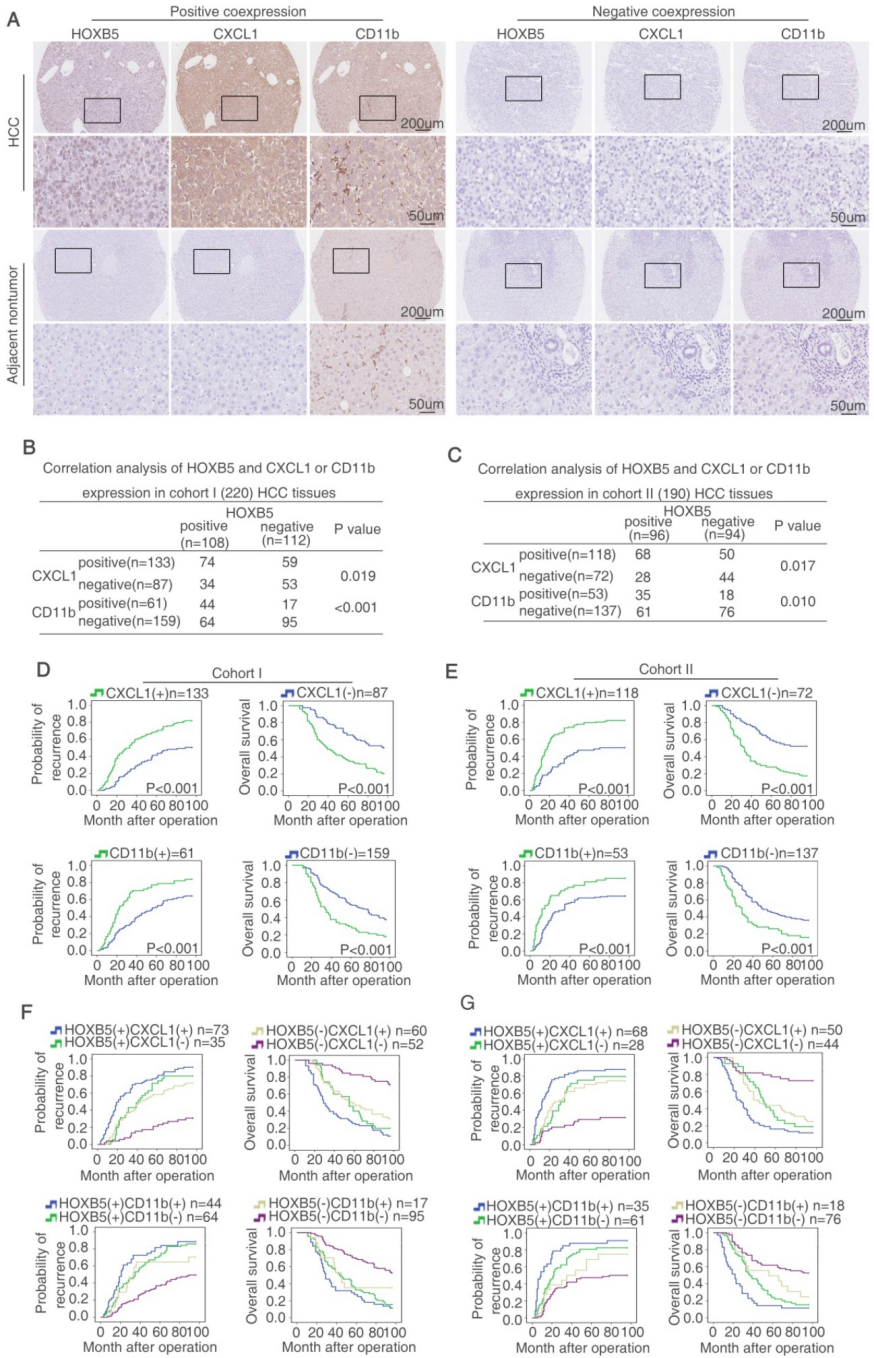
** HOXB5 expression is positively correlated with CXCL1 expression and intratumoral MDSC infiltration in human HCC tissues.** (A) IHC staining showed HOXB5, CXCL1 and CD11b expression in human HCC samples. (B-C) The correlation between HOXB5 and CXCL1 or HOXB5 and CD11b in human HCC tissues was shown in cohort I (B) and cohort II (C). (D-E) Overall survival time and recurrence rate of HCC patients with positive or negative expression of CXCL1 or CD11b were shown in cohort I (D) and cohort II (E). (F-G) Kaplan-Meier analyzed recurrence rate and overall survival time of HCC patients with positive expression of HOXB5/CXCL1 or HOXB5/CD11b.

HOXB5 and CXCL1 expression, and intratumoral MDSCs infiltration (CD11b as marker) were detected by IHC staining in two HCC cohorts. Representative IHC images of HOXB5, CXCL1, and CD11b expression were exhibited in Figure 5A. Moreover, CXCL1 expression was upregulated shown by ELISA (Figure S1L). In both HCC cohorts, HOXB5 expression was positively correlated with CXCL1 expression and intratumoral MDSCs infiltration (Figure 5B-C). Both upregulation of CXCL1 and intratumoral MDSCs infiltration were positively associated with microvascular invasion, poorer differentiation and higher TNM stage (Table S5 and S6). In both cohorts, compared to HCC patients with negative expression of CXCL1, patients with positive expression CXCL1 had reduced overall survival time and increased recurrence rate. Intratumoral MDSCs accumulation indicated unfavorable prognosis (Figure 5D-E). Positive co-expression of HOXB5 and CXCL1 contributed to highest recurrence rate and lowest overall survival time in HCC patients. Similarly, positive co-expression of HOXB5 and intratumoral MDSCs infiltration was correlated with poorest prognosis in HCC patients (Figure 5F-5G). Then we detected the CXCL1 expression using metastatic samples by RT-qPCR and IHC staining. The results showed that CXCL1 expression was upregulated in metastatic samples (Supplementary Figure S5).

### FGF19 activates PI3K/Akt/HIF-1α signaling pathway and upregulates HOXB5 expression

**Figure 6:**
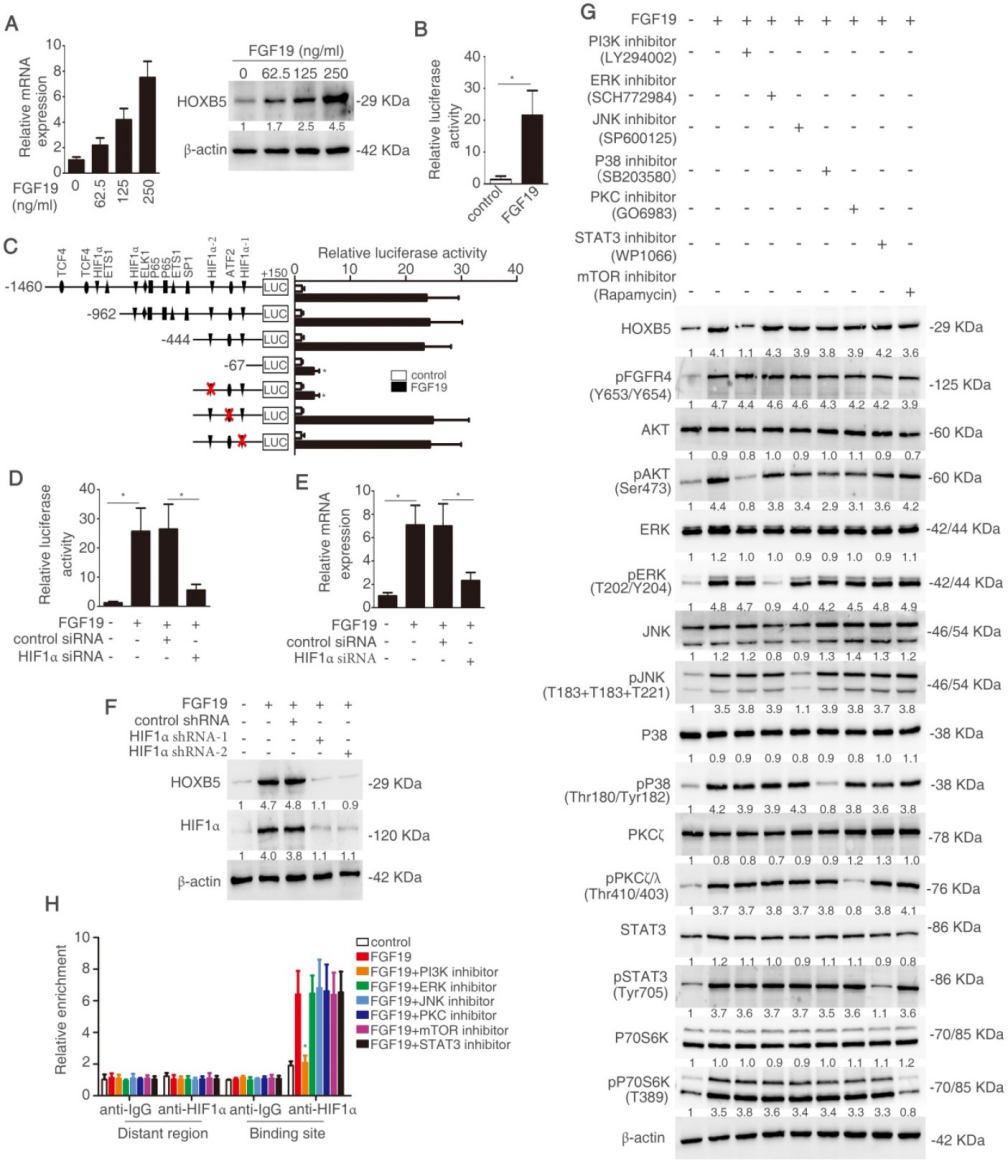
** FGF19 activates PI3K/Akt/HIF-1α signaling pathway and upregulates HOXB5 expression.** (A) Relative mRNA and protein level of HOXB5 were measured by RT-qPCR and Western blotting. (B) HOXB5 promoter luciferase activity was measured after FGF19 stimulation in PLC/PRF/5 cells. (C) Luciferase activity was detected after the transfection of truncated and mutated HOXB5 promoter constructs and FGF19 treatment. (D-F) After transfection of HIF1α shRNA or control shRNA, PLC/PRF/5 cells were then treatment with FGF19. HOXB5 promoter activity (D) and HOXB5 mRNA expression RT-qPCR (E) and protein expression (F) were shown. (G) After pretreatment of signaling pathway inhibitors of PI3K, ERK, JNK, P38, PKC, mTOR and STAT3, PLC/PRF/5 cells were stimulated with FGF19. The expression of HOXB5, pFGFR4 and total or phosphorylated levels of AKT, ERK, JNK, P38, PKC, P70S6K and STAT3 were shown by western blotting. (H) A ChIP assay shown the relative enrichment of HIFIα on HOXB5 promoter when the PLC/PRE/5 cells were stimulated with FGF19 and signaling pathway inhibitors.

Considering the important roles of both FGF19-FGFR4 axis and HOXB5 in metastasis, we hypothesize that whether FGF19 induces HOXB5 expression in HCC cells. PLC/PRF/5 cells with low endogenous FGF19 and HOXB5 expression were treated with different concentrations of FGF19 (Figure S1M). The mRNA and protein levels of HOXB5 were increased in a dose-dependent manner (Figure 6A). Moreover, FGF19 treatment increased the promoter activity of *HOXB5* gene in PLC/PRF/5 cells (Figure 6B). To find the cis-regulatory elements which were involved in FGF19-induced HOXB5 expression, we synthesized the truncated and mutated human *HOXB5* promoter construct between -1460bp and +150bp region. The deletion of *HOXB5* promoter from -1460bp to -444bp did not affect FGF19-induced *HOXB5* promoter transactivation, whereas the deletion of *HOXB5* promoter from -444bp to -67bp dramatically decreased FGF19-induced *HOXB5* promoter transactivation, suggesting that the promoter region of -444bp to 67bp was crucial for FGF19-induced *HOXB5* promoter transactivation. Two potential HIF1α and one ATF2 binding sites were found in this region (Supplementary Table S10). We then directly mutated these binding sites and found that second HIF1α binding site in the *HOXB5* promoter was essential for the increased promoter activity after FGF19 stimulation (Figure 6C). Knockdown of HIF1α decreased *HOXB5* promoter activity and HOXB5 expression in mRNA and protein levels by FGF19 treatment (Figure 6D-F).

FGF19 has been reported to activate MAPK, PI3K, PKC, mTOR, and STAT3 signaling pathway 29. In order to testify which pathway was involved in FGF19-mediated HOXB5 upregulation, PLC/PRF/5 cells were pretreated with these pathway inhibitors. PI3K inhibitor largely inhibited FGF19-induced HOXB5 expression, whereas HOXB5 expression had no significant change under the treatment with other pathway inhibitors (Figure 6G). Furthermore, the ChIP result proved that the binding of HIF1α to the *HOXB5* promoter was blocked by PI3K inhibitor, whereas other pathway inhibitors treatment showed little effect on this binding (Figure 6H). These results evidenced that FGF19-promoted HOXB5 expression was dependent on PI3K/Akt/HIF-1α pathway.

### HOXB5 is essential for FGF19/15-mediated HCC metastasis

**Figure 7:**
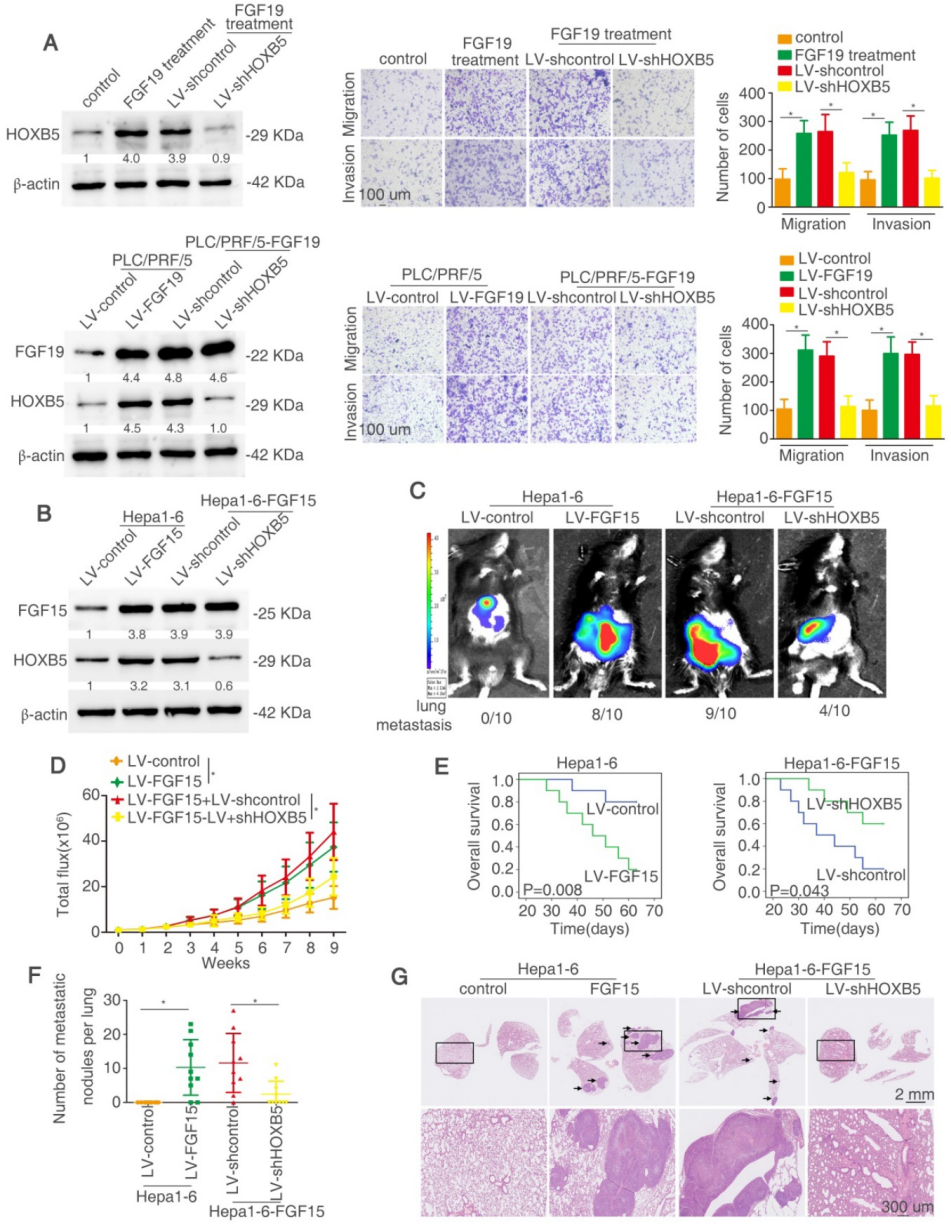
** HOXB5 is essential for FGF19/15-mediated HCC metastasis.** (A) Western blotting showed the HOXB5 and FGF19 expression in different group (left). Transwell showed the ability of migration and invasion of PLC/PRF/5 cells in different group (right). (B) Western blotting showed FGF15 and HOXB5 expression in different group. (C-G) HOXB5 promoted HCC metastasis through upregulating CXCL1. (C-D) The C57BL/6 mice were implanted with the indicated cells in the liver. Bioluminescent images, growth rate and metastatic rate were shown. (E) Survival curve was shown in different groups. (F) Lung metastatic nodules was counted. (G) HE staining showed lung metastatic nodules in different groups. * P < 0.05.

We determined whether HOXB5 is involved in FGF19-mediated HCC metastasis. Firstly, lentivirus LV-shHOXB5 was used to establish stable cell line PLC/PRF/5-shHOXB5 cells and then FGF19 was used to stimulate the cells (Figure 7A). FGF19 treatment increased the migratory and invasive abilities of PLC/PRF/5 cells, whereas knockdown of HOXB5 decreased these effects (Figure 7A upper). We then established stable PLC/PRF/5-FGF19 cells with transfection of lentivirus LV-FGF19 and decreased HOXB5 expression in PLC/PRF/5-FGF19 cells (Figure 7A lower). PLC/PRF/5-FGF19 cells showed increased migratory and invasive abilities compared with the PLC/PRF/5-control cells while downregulation of HOXB5 impaired these effects (Figure 7A). The migrative and invasive abilities of PLC/PRF/5-FGF19 cells were decreased under the treatment of PI3K inhibitor (Figure S6). Next, we used the C57BL/6 mice to perform the *in vivo* metastatic assay. Because of the ortholog of FGF15 in mice and FGF19 in human, we overexpressed FGF15 in mouse HCC cell Hepa1-6 50. We established a stable cell line Hepa1-6-FGF15 and knocked down the HOXB5 expression in these cells (Figure 7B). The *in vivo* metastatic results showed that mice in Hepa1-6-FGF15 group had increased lung metastasis rate and the number of metastatic nodules in the lung and decreased overall survival time compared with mice in control group. However, knockdown of HOXB5 in Hepa1-6-FGF15 cells reversed these results described above (Figure 7C-G). These results evidenced that HOXB5 is essential for FGF19/15-induced HCC metastasis.

We then assessed the clinical importance of HOXB5 and FGF19 or FGFR4 in two independent HCC cohorts. ELISA showed that FGF19 expression was higher in HCC than controls (Supplementary Figure S1L). Representative IHC images were shown in Figure S2A. In both cohorts, HOXB5 expression was positively associated with FGF19 and FGFR4 expression (Figure S2B-C). HCC tissues with elevated expression of FGF19 or FGFR4 were positively correlated with microvascular invasion, poorer differentiation and higher TNM stage (Table S7-S8). In both cohorts, compared to HCC patients with negative expression of FGF19 or FGFR4, patients with positive expression of FGF19 or FGFR4 had higher recurrence rate and shorter overall survival time (Figure S2D-E). Moreover, Kaplan-Meier exhibited that positive co-expression of HOXB5/FGF19 or HOXB5/FGFR4 predicted highest recurrence rate and shortest survival time of HCC patients (Figure S2F-G).

### Combined treatment of FGFR4 inhibitor BLU-554 and CXCR2 inhibitor SB265610 dramatically decreases HOXB5-mediated HCC metastasis

**Figure 8:**
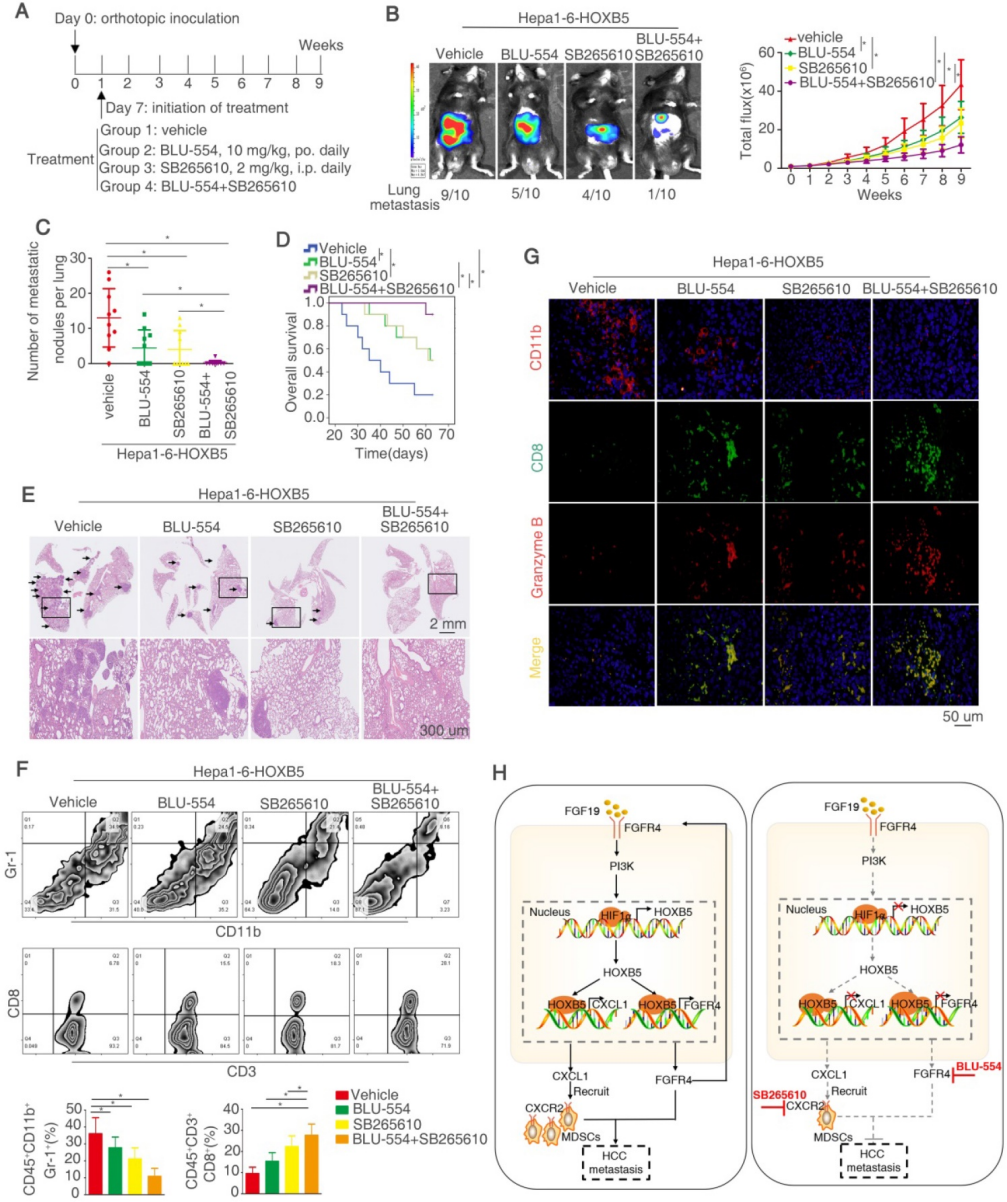
** Combined treatment of FGFR4 inhibitor BLU-554 and CXCR2 inhibitor SB265610 dramatically decreased HOXB5-driven HCC metastasis.** (A) The diagram of *in vivo* treatment in C57/BL mice. One week after injection of PLC-PRF/5-HOXB5 cells, mice in four group were treated with vehicle, BLU-554 or SB265610 or combined treatment respectively. (B-E)* In vivo* assays showed that combined treatment of FGFR4 and CXCR2 inhibitors can almost block HCC metastasis totally. (B) Representative Bioluminescence images, growth rate and lung metastasis rate were shown in different groups. (C) Metastatic lung nodules were shown. (D) Survival curve was shown in different mice. (E) HE staining shown lung metastatic nodules in different mice groups. (F) Flow cytometry showed the percent of MDSCs and CD8^+^T cells. (G) IF showed the infiltration of MDSCs and CD8^+^T cell in different groups. (H) A schematic diagram illustrated the importance of FGF19-HOXB5 signaling in HCC metastasis. FGF19-FGFR4 signaling upregulated HOXB5 expression through PI3K/Akt/HIF-1α pathway. HOXB5 promoted HCC metastasis through transactivating FGFR4 and CXCL1. Combined FGFR4 inhibitor BLU-554 and CXCR2 inhibitor SB265610 almost abolished HOXB5-induced HCC metastasis. * P < 0.05.

Our above works have proved that FGF19-induced HOXB5 upregulation promoted HCC metastasis through transactivating FGFR4 and CXCL1 expression. Therefore, we determined whether combined treatment of BLU-554, a highly selective, small-molecule inhibitor of FGFR4 30 and CXCL1/CXCR2 pathway inhibitors SB265610 32 had any effect on HOXB5-induced HCC metastasis. In order to investigate this hypothesis, we designed the *in vivo* experiment (Figure 8A). These treatments had no significantly toxicity in these mice (Figure S1N). The *in vivo* metastatic assay demonstrated that BLU-554 or SB265610 treatment alone partially impaired the lung metastasis rate and metastatic nodules in the lung and partially prolonged the overall survival time of mice in Hepa1-6-HOXB5 group, whereas combination of BLU-554 and SB265610 significantly decreased the lung metastasis rate and lung metastatic nodules and largely prolonged survival time compared with control or single agent treatment (Figure 8B-E). We then analyzed the number of MDSCs and CD8^+^T cells in the orthotopic tumors by flow cytometry. The results exhibited that combined treatment largely decreased the infiltration of MDSCs while increased accumulation of CD8^+^T cells (Figure 8F). Moreover, IF staining showed that combined treatment reduced the amount of MDSCs and improved the activity of CD8^+^T cells in the orthotopic tumors (Figure 8G). These studies suggested that the combined targeting FGFR4 and MDSCs significantly suppressed HOXB5-mediated HCC metastasis.

## Discussion

Because of early relapse and metastasis, a better understanding of the oncogenic genes may help to acquire knowledge of molecular profiling in HCC metastasis and develop more potent combination-based therapies 51. In this study, HOXB5 expression was significantly upregulated in metastatic HCC tissues than in primary HCC tissues. The loss of tumor encapsulation, microvascular invasion, poor differentiation and a higher TNM stage was found in HCC tissues with elevated HOXB5 expression. HOXB5 expression was an independent predictor for recurrence rate and overall survival time in HCC patients presented by multivariate analysis. Furthermore, we found that HOXB5 promoted the migration and invasion of HCC cells *in vitro* and induced metastasis *in vivo* and knockdown of HOXB5 expression impaired these effects. These evidences demonstrated the importance of HOXB5 in promoting HCC metastasis.

FGFR4 maintains liver homeostasis through regulating lipid, glucose and bile acids metabolism. FGFR4 expression is significantly upregulated and functions as an oncogene in numerous cancers including HCC 52. FGFR4 promotes proliferation and metastasis of HCC, and elevated expression of FGFR4 is involved in sorafenib resistance 29, 52. The administration of FGFR4 specific inhibitors significantly suppressed HCC progression, indicating the oncogenic driver role of FGFR4 in HCC 30, 53. CXCL1, an important ligand of CXCR2, which promotes cancer chemoresistance, progression and metastasis 54, recruits MDSCs to tumor site to induce immune suppression 55. Several recent studies report that CXCL1-CXCR2 axis promotes HCC progression and metastasis through the recruitment of MDSCs to the tumor sites 2, 32, 36, 56. These evidences indicate the crucial function of FGFR4 and CXCL1 in HCC metastasis. In this study, we demonstrated that overexpression of HOXB5 transactivated FGFR4 and CXCL1 expression through direct binding to their promoters. Knockdown of FGFR4 and CXCL1 decreased HOXB5-mediated HCC metastasis, while ectopic overexpression of FGFR4 and CXCL1 rescued the impaired HCC metastasis induced by HOXB5 knockdown. These studies indicated that HOXB5 promoted HCC metastasis through regulating its target genes FGFR4 and CXCL1 expression. Moreover, overexpression of HOXB5 in HCC cells promoted the migration and infiltration of MDSCs to tumor sites through CXCL1-CXCR2 axis. Either depletion of MDSCs by anti-Gr1 or the administration of CXCR2 inhibitor SB265610 impaired HOXB5-mediated HCC metastasis. In human HCC tissues, HOXB5 expression was largely associated with CXCL1 expression and intratumor MDSCs infiltration (marked by CD11b staining), and patients with positive co-expression of HOXB5/CXCL1 or HOXB5/CD11b predicted the highest recurrence rate and the lowest overall survival time. These studies demonstrated that CXCL1-CXCR2 induced MDSCs infiltration is important for HOXB5-mediated HCC metastasis.

Emerging data implicates that FGF19 is overexpressed in HCC patients due to several reasons including genomic amplification on chromosome 11q13.3, and epigenetic upregulation of FGF19^30^. FGF19/FGFR4 axis plays an import role in promoting HCC progression and metastasis through activating several oncogenic signaling pathways, such as β-catenin and STAT3 26. In this study, FGF19 upregulated HOXB5 expression through the PI3K/AKT-HIF1α signaling pathway. HOXB5 directly bound to *FGFR4* promoter and upregulated FGFR4 expression, which formed a FGF19-HOXB5-FGFR4 positive feedback loop in HCC cells. Moreover, overexpression of FGF15 (an analog of FGF19 in mouse) significantly promoted HCC metastasis, whereas knockdown of HOXB5 dramatically decreased FGF15-mediated HCC metastasis in immunocompetent mice. These studies indicated that FGF19/15-HOXB5-FGFR4 loop played an important role in promoting HCC metastasis. It is noteworthy that HOXB5 can promote metastasis through different molecular mechanisms in other cancer types. For instance, HOXB5 can facilitate the metastasis of head and neck squamous carcinoma cells through the EGFR/Akt/Wnt/β-catenin signaling axis 45. While our work illustrated FGFR4 was an important downstream player for HCC metastasis mediated by HOXB5 overexpression, other signaling pathways may also contribution to this process.

The development of molecular targeted therapies in cancer has proved that targeting sub-classification of patients with a molecular alteration provides better response 57. To design pharmacological strategy against the positive feedback loop reported in our study, we focused on FGFR4 and CXCR2 inhibitors. BLU-554, a highly selective, small-molecule inhibitor of FGFR4, exhibits robust antitumor effect *in vitro* and *in vivo* 30, 53. However, the objective response rate (ORR) is still low 57. SB265610, an inhibitor of CXCR2, has been reported to inhibit the recruitment and infiltration of MDSCs in HCC 32. In our study, we hypothesized that combination of FGFR4 inhibitor BLU-554 and CXCR2 inhibitor SB265610 had any effect on HCC metastasis enhanced by HOXB5 overexpression. Our *in vivo* data showed that combined treatment of both inhibitors dramatically inhibited HOXB5-mediated HCC metastasis compared with control or single agent alone. These results provided a new combinational therapeutic strategy to inhibit HOXB5-induced HCC metastasis.

In conclusion, we demonstrated that overexpression of HOXB5 induced by FGF19-FGFR4 signaling contributed to HCC metastasis through the upregulation of FGFR4 and CXCL1 expression. Combined targeting FGFR4 and MDSCs largely suppressed HOXB5-mediated HCC metastasis. Therefore, HOXB5 was a potential prognostic biomarker in HCC patients and targeting the oncogenic FGF19-HOXB5-FGFR4 loop may provide a promising treatment strategy for HOXB5-upregulated HCC subpopulation.

## Supplementary Material

Supplementary figures and tables.

## Abbreviations

BLI: bioluminescent imaging
ChIP: chromatin immunoprecipitation analysis
CXCL1: C-X-C motif chemokine ligand 1
ELISA: enzyme linked immunosorbent assay
FGF15: fibroblast growth factor 15
FGF19: fibroblast growth factor 19
FGFR4: fibroblast growth factor receptor 4
HCC: hepatocellular carcinoma
ERK: extracellular regulated protein kinase
JNK: Jun N-terminal kinase
PI3K: phosphoinositide 3-kinase
PKC: protein kinase C
mTOR: mammalian target of rapamycin
STAT3: signal transducer and activator of transcription 3
HIF1α: Hypoxia inducible factor 1 subunit alpha
HOXB5: homeobox B5
H&E: Hematoxylin and eosin
IF: immunofluorescence
IHC: immunohistochemistry
MDSCs: myeloidderived suppressor cells
mRNA: messenger RNA
shRNA: short hairpin RNA
TAMs: tumor-associated macrophages
TNM: tumor-nodule-metastasis
