# Supplementary material for: Homeobox B5 promotes metastasis and poor prognosis in Hepatocellular Carcinoma, via FGFR4 and CXCL1 upregulation
Source: Theranostics. 2021 Mar 31;11(12):5759–77. doi: 10.7150/thno.57659 (PMC8058721; doi:10.7150/thno.57659)
Supplement: Supplementary file 1 — Supplementary figures and tables. [file thnov11p5759s1.pdf]

Supplementary materials

Materials and Methods

Cell culture

Immortalized liver cell lines (HL-7702), HCC cells Huh6 and Hepa1-6 were purchased from the Institute of Biochemistry and Cell Biology, Chinese Academy of Science, China. Human HCC cells (HepG2, Huh-7, Hep3B, PLC/PRF/5, SNU423, SNU398, SNU387 and SNU878) were purchased from the American Type Culture Collection. Additional human HCC cells (MHCC97H, HCCLM3, and HCCLM6) were kindly provided by Dr. Tang ZY (Liver Cancer Institute, Zhongshan Hospital, Fudan University, Shanghai, China). Cells were cultured in Dulbecco's Modified Eagle Medium (DMEM) at 37°C in a 5% CO_2 incubator. The medium was supplemented with 10% FBS, 100μg/ml penicillin, and 100μg/ml streptomycin. These above cell lines were authenticated by short tandem repeats (STRs) DNA profiling. All cells were tested for mycoplasma contamination before use with the Universal Mycoplasma Detection Kit (ATCC 30-1012K) and were not contaminated by mycoplasma.

Patients and follow-up

This study was approved by the Ethics Committee of Tongji Medical College. All patients provided full consent for the study. Cohort I included 220 adult patients with HCC who underwent curative resection between 2003 and 2005 at the Tongji Hospital of Tongji Medical College (Wuhan, China). Cohort II included 190 adult patients with

HCC who underwent curative resection between 2006 and 2008 at the Tongji Hospital of Tongji Medical College (Wuhan, China). A preoperative clinical diagnosis of HCC was based on the diagnostic criteria of the American Association for the Study of Liver Diseases. The inclusion criteria were as follows: (a) distinctive pathologic diagnosis; (b) no preoperative anticancer treatment or distant metastases; (c) curative liver resection; and (d) complete clinicopathologic and follow-up data. The differentiation statuses were graded according to the method of Edmondson and Steine. The pTNM classification for HCC was based on The American Joint Committee on Cancer/International Union Against Cancer staging system (6th edition, 2002). Follow-up data were summarized at the end of December 2013 (Cohort I) and December 2016 (Cohort II, range 4-96 months) respectively. The patients were evaluated every 2-3 months during the first 2 years and every 3-6 months thereafter. All follow-up examinations were performed by physicians who were blinded to the study. During each check-up, the patients were monitored for tumor recurrence by measuring the serum AFP levels and by performing abdominal ultrasound examinations. A computed tomography and/or magnetic resonance imaging examination was performed every 3-6 months, together with a chest radiographic examination. The diagnostic criteria for HCC recurrence were the same as the preoperative criteria. The time to recurrence and overall survival were the primary endpoints. The time to recurrence was calculated from the date of resection to the date of a diagnosis with tumor recurrence. The overall survival was calculated from the date of resection to the date of death or of the last follow-up.

In addition, 10 normal liver tissues, 50 pairs of fresh HCC tissues and adjacent nontumor tissue samples and 30 pairs of fresh metastatic and matched primary HCC tissues and adjacent nontumor tissue samples were collected after surgical resection and were used to detect the mRNA levels of HOXB5.

Reagents

PI3K inhibitor LY294002, ERK inhibitor SCH772984, JNK inhibitor SP600125, PKC inhibitor GO6983, mTOR Rapamycin, STAT3 inhibitor WP1066, BLU-5543 and SB265610 were purchased from MedChemExpress. All the reagents were used according to the manufacturer's instruction.

Plasmid construction

Plasmid construction was performed according to standard procedures as outlined in our previous study[1]. The primers are presented in Supplementary Table S9. For example, the FGFR4 promoter construct, (-1781/+138) FGFR4, was generated from human genomic DNA. This construct corresponded to the sequence from -1781/+138 from the 5'-flanking region of the human FGFR4 gene. It was generated with forward and reverse primers incorporating KpnI and MluI sites at the 5' and 3'-ends, respectively. The polymerase chain reaction (PCR) product was cloned into the KpnI and MluI sites of the pGL3-Basic vector (Promega). The 5'-flanking deletion constructs of the FGFR4 promoter, (-1335/+138) FGFR4 and (-765/+138) FGFR4 were similarly generated using the (-1781/+138) FGFR4 construct as the

template. The HOXB5 binding sites in the FGFR4 promoter were mutated using the QuikChange II Site-Directed Mutagenesis Kit (Stratagene). The constructs were confirmed by DNA sequencing. Other promoter constructs were cloned in the same manner.

Construction of lentivirus and stable cell lines

Lentiviral vectors encoding shRNAs were generated using PLKO.1-TRC (Addgene) and designated as LV-shHOXB5, LV-shFGFR4, LV-shCXCL1 and LV-shcontrol. "LV-shcontrol" is a non-target shRNA control. The vector "pLKO.1-puro Non-Target shRNA Control Plasmid DNA" (purchased from Sigma, SHC016) contains an shRNA insert that does not target any known genes from any species. The shRNA sequences can be found in Supplementary Table S10. Lentiviral vectors encoding the human HOXB5, FGFR4, CXCL1 and FGF19 genes were constructed in pLV-puro or pLV-neo (Addgene) and designated as LV-HOXB5, LV-FGFR4, LV-CXCL1 and LV-FGF19. An empty vector was used as the negative control and was designated as LV-control. The lentivirus and cell infection were produced according to the lentiviral vector protocol recommended by Addgene. Briefly, the lentiviral plasmid and packaging plasmids pMD2. G and psPAX2 (Addgene plasmid #12259 and #12260) were transfected into HEK-293T cells with transfection reagent (Lipofectamine®3000, Thermo Fisher Scientific) and OPTI-MEM media (Invitrogen, Waltham, MA, USA). The lentiviruses were harvested twice on days 4 and 5. Viruses were filtered with a 0.45-μm filter and stored at -80 °C. Lentiviral infection of target cells were performed

in cell culture media with puromycin or G418. Selected pools of knockdown and over-expressing cells were used for the following experiments.

Transient transfection

The cells were plated at a density of 1×10^5 cells/well in a 24-well plate. After 12-24 hours, the cells were co-transfected with 0.6μg of the expression vector plasmids, 0.18 μg of the promoter reporter plasmids and 0.02μg of the pRL-TK plasmids using Lipofectamine 2000 (Invitrogen, USA) according to the manufacturer's instructions. After 5 h of transfection, the cells were washed and allowed to recover overnight in fresh medium supplemented with 1% FBS for 48 h. Serum-starved cells were used for the assay.

Western blotting analyses

Briefly, proteins were separated by SDS-PAGE and transferred to polyvinylidene difluoride membrane. 5% BSA was used to block the nonspecific binding site for two hours in room temperature. The membranes were incubated with primary antibodies overnight at 4 °C. Western blotting of β-actin on the same membrane was used as a loading control. The membranes were washed with TBST for 3 times with ten minutes each and then incubated with an HRP-conjugated secondary antibody. Proteins were visualized using an ImmobilonTM Western Chemiluminescent HRP substrate (Millipore, USA).

The primary antibodies used in western blotting were listed below.

Antibodies	Source
anti-β-actin	Proteintech, 60008-1-Ig
anti-HOXB5	Abcam, ab229345
anti-HOXB5	Invitrogen, PA5-36381
anti-FGFR4	R&D, MAB6852
anti-FGFR4	Proteintech Group, 11098-1-AP
anti-HIF1α	Abcam, ab1
anti-ERK1/2	Cell Signaling Technology, #9102
anti-p-ERK1/2(T202/Y204)	Cell Signaling Technology, #4370
anti-JNK	Abcam, ab179461
anti-JNK(phosphoT183+T183+T221))	Abcam, ab124956
anti-AKT	Cell signaling technology, #4685
anti-pAKT (Ser-473)	Cell signaling technology, #4060
anti-PKCζ	Cell Signaling Technology, #9368
anti-p-PKCζ(Thr410/403)	Cell Signaling Technology, #9378
anti-p70S6K	Cell Signaling Technology, #2708
anti-p-p70S6K(T389)	Cell Signaling Technology, #9234
anti-STAT3	Cell Signaling Technology, #9139
anti-p-STAT3 (Tyr705)	Cell Signaling Technology, #9145
anti-FGF19	Cell Signaling Technology, #83348
anti-FGF15	Abcam, ab229630

Construction of tissue microarrays and immunohistochemistry

HCC samples and the corresponding adjacent liver tissues were used to construct a tissue microarray (Shanghai Biochip Co., Ltd. Shanghai, China). IHC was performed on 4-μm-thick, routinely processed paraffin-embedded sections. Briefly, the tissue sections were deparaffinized after baking at 60 °C for an hour. Endogenous peroxidase activity was blocked by 3% (vol/vol) hydrogen peroxide in methanol for 12 min and washes with phosphate-buffered saline (PBS). Then the slides were immersed in 0.01 mol/L citrate buffer solution (pH 6.0) and placed in a microwave oven for 30 min. After washed with PBS, the sections were incubated with the primary antibody diluted in PBS containing 1% (wt/vol) bovine serum albumin in 4 °C for overnight. The tissue microarrays were stained for HOXB5 (Abnova, PAB30143), CXCL1 (LifeSpan Biosciences, LS-B2843), CD11b (Abcam, ab133357), FGFR4 (R&D, MAB6852), FGF19(cell signaling technology, #83348). Negative controls were performed by replacing the primary antibody with preimmune mouse serum. After washed with PBS, the sections were treated with a peroxidase-conjugated second antibody (Santa Cruz) for 30 min at room temperature and then washed with PBS. Reaction product was visualized with diaminobenzidine for 2 min. Images were obtained under a light microscope (Olympus, Japan) equipped with a DP70 digital camera.

Analyses were performed by two independent observers who were blinded to the clinical outcome. The immunostaining intensity was scored on a scale of 0 to 3: 0 (negative), 1 (weak), 2 (medium) or 3 (strong). The percentage of positive cells was

evaluated on a scale of 0 to 4: 0 (negative), 1 (1%-25%), 2 (26%-50%), 3 (51%-75%), or 4 (76%-100%). The final immuno-activity scores were calculated by multiplying the above two scores, resulting an overall score which range from 0~12. Each case was ultimately considered "negative" if the final score ranges from 0~3, and "positive" if the final score ranges from 4~12 as described previously[1].

Antibodies used in Immunofluorescence

Antibodies	Source
anti-CD11b	Abcam, ab133357
Anti-CD8a	Invitrogen, 4SM15
Anti-Granzyme B	R&D, AF1865

Antibodies used in Flow Cytometric Analysis

Antibodies	Source
anti-CD45	BD, 559864
anti-CD11b	BD, 564454
anti-Gr-1	eBioscience, 12-5931-62
anti-CD3	BD, 555275
anti-CD8a	BD, 564459
7-AAD	Absin, abs9104

Quantitative Real-time PCR (RT-qPCR)

The RNeasy Plus Mini Kit (50) kit (Qiagen, Hilden, Germany) was used to extract total RNA, which was then reverse transcribed with the Advantage RT-for-PCR Kit

(Qiagen) in accordance with the manufacturer's protocols. The target sequence was amplified with real-time PCR with the SYBR Green PCR Kit (Qiagen). The cycling parameters used were 95 °C for 15 s, 55-60 °C for 15 s, and 72 °C for 15 s for 45 cycles. Melting curve analyses were performed, and Ct values were determined during the exponential amplification phase of real-time PCR. SDS 1.9.1 software (Applied Biosystems, Massachusetts, USA) was used to evaluate amplification plots. The $2^{-\Delta\Delta Ct}$ method was used to determine relative fold changes in target gene expression in cell lines, which was normalized to expression levels in corresponding control cells (defined as 1.0). The equation used was $2^{-\Delta\Delta Ct}$ ($\Delta Ct = \Delta Ct^{target} - \Delta Ct^{GAPDH}$; $\Delta\Delta Ct = \Delta Ct^{expressing\ vector} - \Delta Ct^{control\ vector}$). When calculating relative expression levels in surgically extracted HCC samples, relative fold changes in target gene expression were normalized to expression values in normal liver tissues (defined as 1.0) using the following equation: $2^{-\Delta\Delta Ct}$ ($\Delta\Delta Ct = \Delta Ct^{tumor} - \Delta Ct^{nontumor}$). All experiments were performed in duplicate. The primer sequences were listed in Supplementary Table S9.

Luciferase reporter assays

Luciferase activity was detected using the Dual Luciferase Assay (Promega, USA) according to the manufacturer's instructions. The transfected cells were lysed in culture dishes and the lysates were centrifuged at maximum speed for 1 min. Relative luciferase activity was determined using a ModulusTM TD20/20 Luminometer (Turner Biosystems, USA) and the transfection efficiencies were normalized

according to the Renilla activity.

Enzyme-Linked Immunosorbent Assay

CXCL1 protein in conditioned medium was detected by using the Human CXCL1/GRO alpha DuoSet ELISA Kit (DY275, R&D Systems) accordance with research paper[2].

The human tumor metastasis or human Cytokines & Chemokines RT^2 profiler PCR array

PLC/PRF/5 cells were divided into two groups, namely PLC/PRF/5-control and PLC/PRF/5-HOXB5. Similarly, MHCC97H cells were divided into two groups, namely MHCC97H-shcontrol and MHCC97H-shHOXB5. RNA extraction, DNase treatment, and RNA cleanup were performed according to the manufacturer's protocol (Qiagen). The cDNA of each group was synthesized using the RT^2 First Strand Kit (Qiagen). Gene expression profiling of PLC/PRF/5-control and PLC/PRF/5- HOXB5 cells or MHCC97H-shcontrol and MHCC97H-shHOXB5 were conducted using the Human tumor metastasis RT^2 Profiler PCR Array and Human Cytokines & Chemokines RT^2 Profiler PCR Array. The cDNA synthesis reaction was mixed with 2× RT^2 qPCR SYBR Green Mastermix and ddH_2O, and then dispensed to the PCR array 96-well plate (25 μL/well). A 2-step cycling program was performed using the Bio-Rad CFX96. Data normalization was done by correcting all Ct values based on the average Ct values of several housekeeping genes present on array. Each assay was

conducted in triplicate.

In vitro invasion and migration assay

Transwell inserts were placed inside 24-well cell culture plates (8 μm pore size; Corning, USA). The upper chamber was coated with 60 μL of Matrigel (Corning, 200 mg/mL) and dried overnight at 37°C in a 5% CO2 incubator for invasion assays. For the migration and invasion assays, 5×10^4 and 1×10^5 cells, respectively, were plated in the top chamber, and the lower chamber was filled with 500 μL of complete medium. After incubation for 24 h, the cells on the upper surface of the membranes were removed by swabbing, and the cells on the lower surface were fixed with 4 % paraformaldehyde for 10 min and stained with 0.1% crystal violet for 15 min. The average number of cells in five fields per membrane was counted on three inserts.

MDSCs migration assay

MDSCs were isolated by Myeloid-Derived Suppressor Cell Isolation Kit (Miltenyi Biotech) according to the manufacturer's instructions. MDSCs were resuspended and added to the upper chamber of 8-μm pore Transwell inserts (Corning) for 2 h to attach to the membrane. The Transwells were then moved to 24-well plates containing 500μl cell conditioned medium and incubated at 37°C for 24 h. Cells were fixed in 4% paraformaldehyde and stained with 0.1% crystal violet. Migrated cells in the lower chamber were quantified using 10 random fields. Three independent experiments were performed for each assay.

Bioluminescent imaging of metastatic models *in vivo*.

The *in vivo* tumor formation and metastases were monitored using the bioluminescence. For *in vivo* signal detection, D-luciferin (Perkin-Elmer) at 100 mg/kg was injected intraperitonially into the mice. Bioluminescent images were captured using a Lago X optical imaging system Imaging System (SI Imaging). The grow rate graph was shown by GraphPad Prism 8.0 software. At the 9 weeks, the mice were sacrificed and the lungs and livers were collected for histological examination.

References

1. Chen J, Du F, Dang Y, Li X, Qian M, Feng W, et al. Fibroblast Growth Factor 19-Mediated Up-regulation of SYR-Related High-Mobility Group Box 18 Promotes Hepatocellular Carcinoma Metastasis by Transactivating Fibroblast Growth Factor Receptor 4 and Fms-Related Tyrosine Kinase 4. Hepatology. 2020; 71: 1712-31.
2. Li YM, Liu ZY, Wang JC, Yu JM, Li ZC, Yang HJ, et al. Receptor-Interacting Protein Kinase 3 Deficiency Recruits Myeloid-Derived Suppressor Cells to Hepatocellular Carcinoma Through the Chemokine (C-X-C Motif) Ligand 1-Chemokine (C-X-C Motif) Receptor 2 Axis. Hepatology. 2019; 70: 1564-81.

Supplementary figure legends

Figure S1

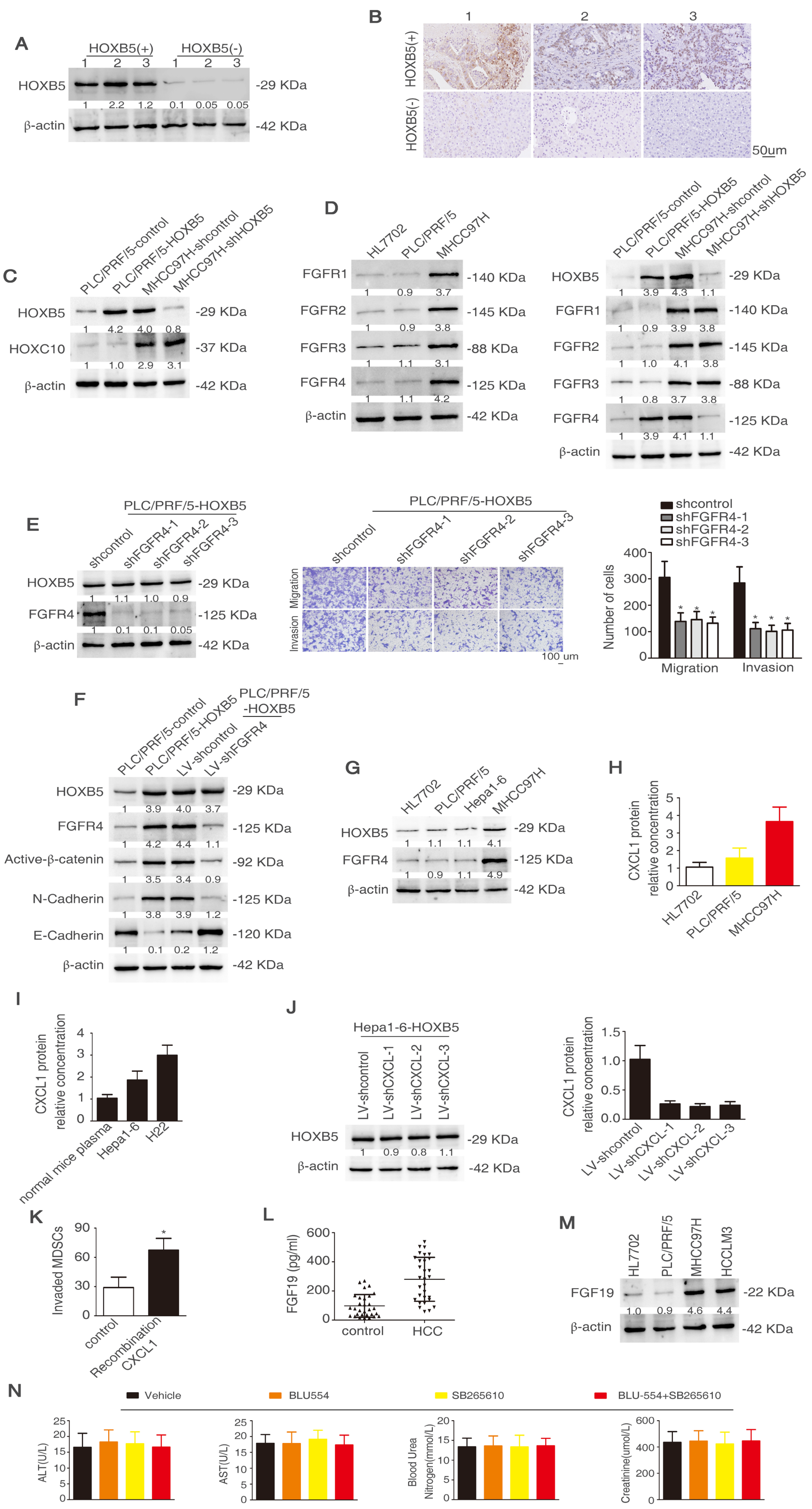

Figure S1. Figure legends

(A-B) Western blotting and IHC were used to show HOXB5 expression and HOXB5 staining.

(C) Western blotting showed the HOXB5 and HOXC10 expression.

(D) Western blotting was used to show HOXB5 and FGFR family expression.

(E) Western blotting showed the expression of FGFR4 when PLC/PRF/5-HOXB5 cells transfected with shFGFR4. Tanswell was used to demonstrate the role of FGFR4 on the migrative and invasive abilities of PLC/PRF/5-HOXB5 cells.

(F) Western blotting showed the active-β-catenin and EMT expression.

(G) The expression of HOXB5 in HL7702, PLC/PRF/5, Hepa1-6 and MHCC97H.

(H) ELISA was used to show the CXCL1 expression in HL7702, PLC/PRF/5 and MHCC97H cells.

(I) ELISA was used to show the CXCL1 expression in normal mice plasm, Hepa1-6 and H22.

(J) Western blotting and ELISA were used to detect the CXCL1 expression.

(K) MSDCs treated with or without CXCL1, the migratory ability of MDSCs was shown. Bars represented the means ± SD of 3 independent experiments.

(L) FGF19 expression in healthy donor and HCC patients.

(M) Western blotting showed the FGF19 expression in HL7702, PLC/PRF/5, MHCC97H and HCCLM3 cells.

(N) The effects of BLU554 and/or SB265610 on kidney and liver functions in mice with Hepa-1-6-HOXB5 tumors after treatment at endpoint.

Figure S2

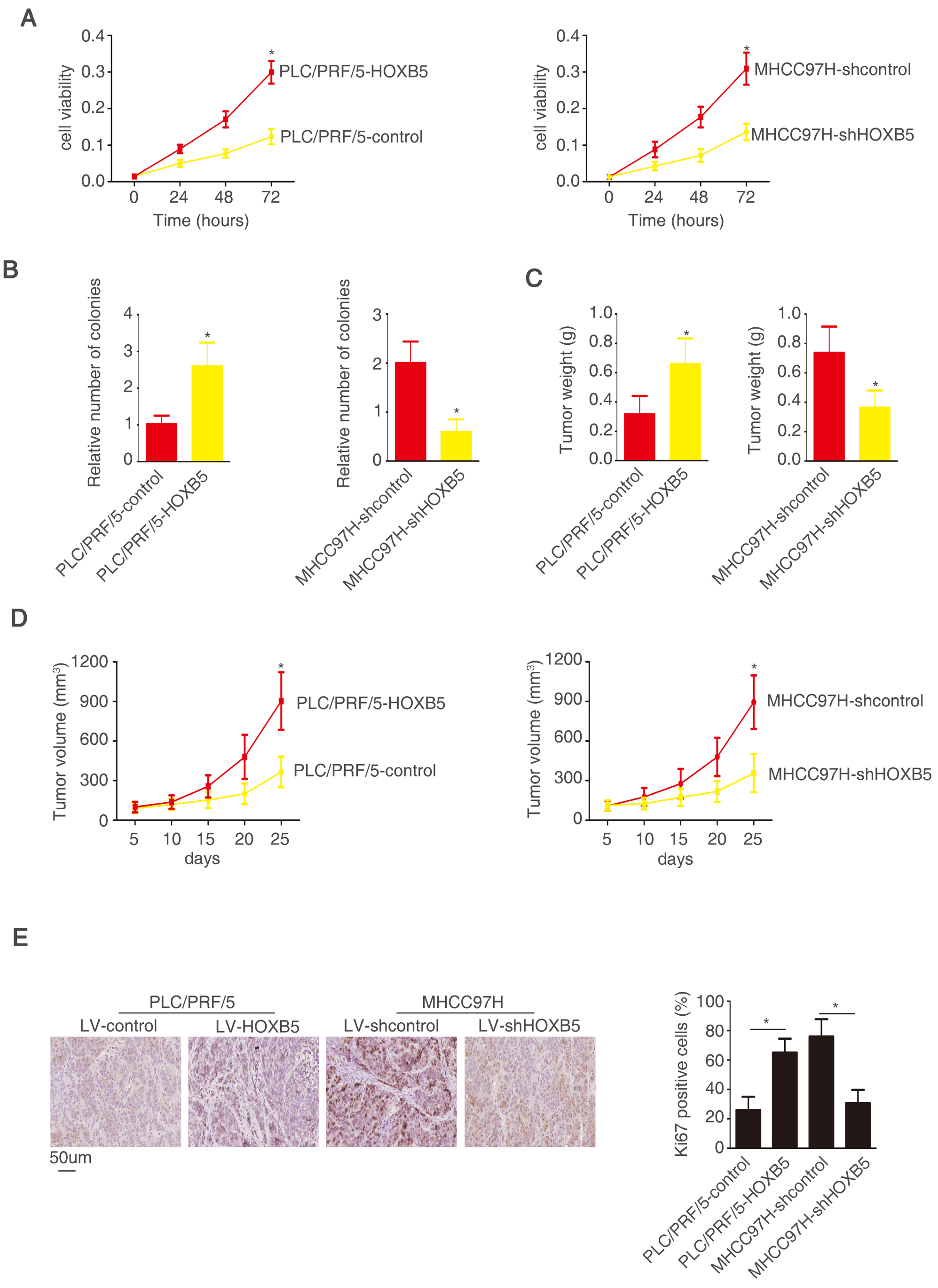

Figure S2. HOXB5 promotes HCC proliferation.

(A) Cell proliferation of the indicated HCC cells was assessed by a Cell Counting Kit-8 (CCK8) assay. The data were expressed as the mean ± S.D. of three experiments.

(B) Indicated cells were subjected to colony formation assays. Five hundred cells were seeded into dishes with medium. Two weeks later, the colonies >40 um were counted and their numbers were compared. Statistical comparison of indicated groups was performed. The data were expressed as the mean ± S.D. of three experiments.

(C) Tumor weight of the indicated groups were detected.

(D) Tumor growth of the indicated HCC cells was investigated by subcutaneous xenograft tumor models. Each experimental group contained 10 nude mice. Data shown the growth curves of tumors in the indicated groups.

(E) Representative Ki67 staining images of tumors from different groups. Scale bars represented 100μm. The percentage of Ki67-positive cells in the indicated tumors was shown.

Figure S3

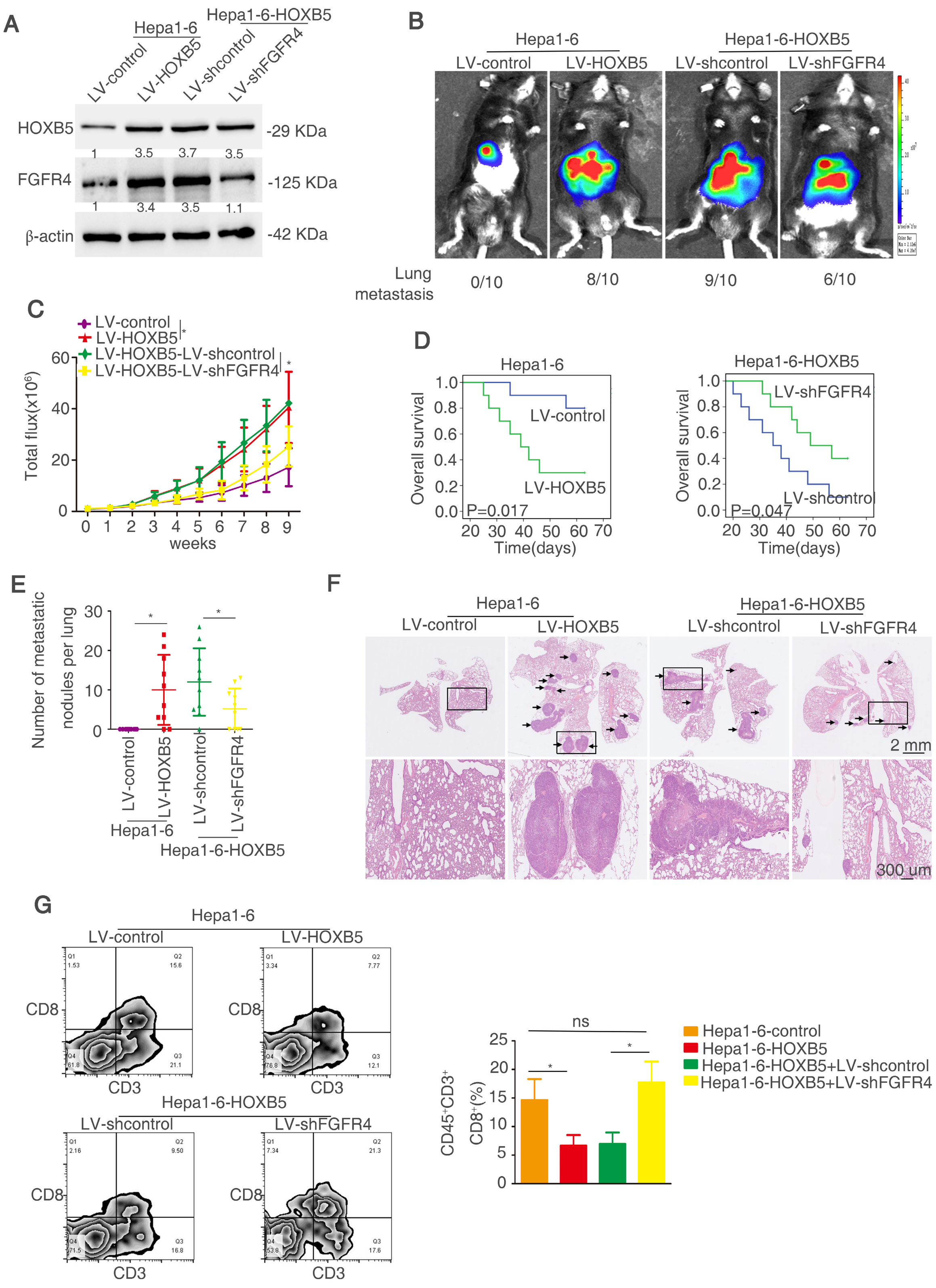

Figure S3. Knockdown of FGFR4 partially inhibits HOXB5-promoted HCC

metastasis in immunocompetent mice.

(A) Western blotting was used to analyze the protein expression of HOXB5 and FGFR4 in indicated HCC cells.

(B-F) In vivo assays showed the function of FGFR4 in HOXB5-promoted HCC metastasis in immunocompetent mice. (B-C) The C57BL/6 mice were implanted with the indicated cells in the liver. Representative bioluminescent images, growth rate and lung metastasis rate in the different groups were shown. (D) Overall survival time of the treated C56BL/6 mice in different groups was shown. (E) the metastatic nodules were counted. (F) Representative HE staining images of lung metastatic nodules in different groups were shown.

(G) CD8+T cells were analyzed by flow cytometry.

Figure S4

A

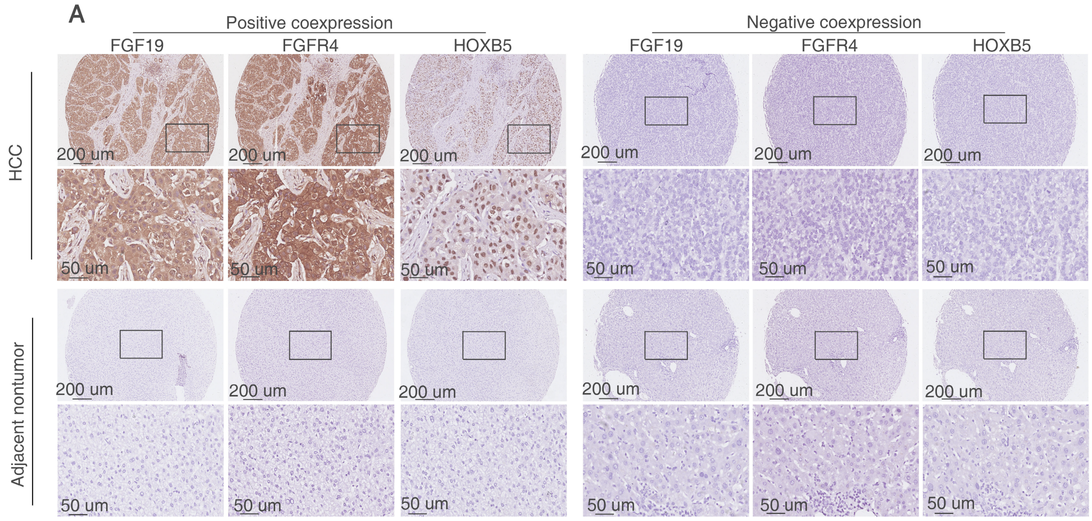

B Correlation analysis of HOXB5 and FGF19 or FGFR4 expression in cohort I (220) HCC tissues

		HOXB5 positive (n=108)	HOXB5 negative (112)	P value
FGF19	positive(n=111)	64	47	0.011
	negative(109)	44	65	
FGFR4	positive(n=117)	68	49	0.005
	negative(n=103)	40	63	

C Correlation analysis of HOXB5 and FGF19 or FGFR4 expression in cohort II (190) HCC tissues

		HOXB5 positive (n=96)	HOXB5 negative (n=94)	P value
FGF19	positive(n=98)	60	38	0.004
	negative(n=92)	36	56	
FGFR4	positive(n=100)	61	39	0.004
	negative(90)	35	55	

D E F G

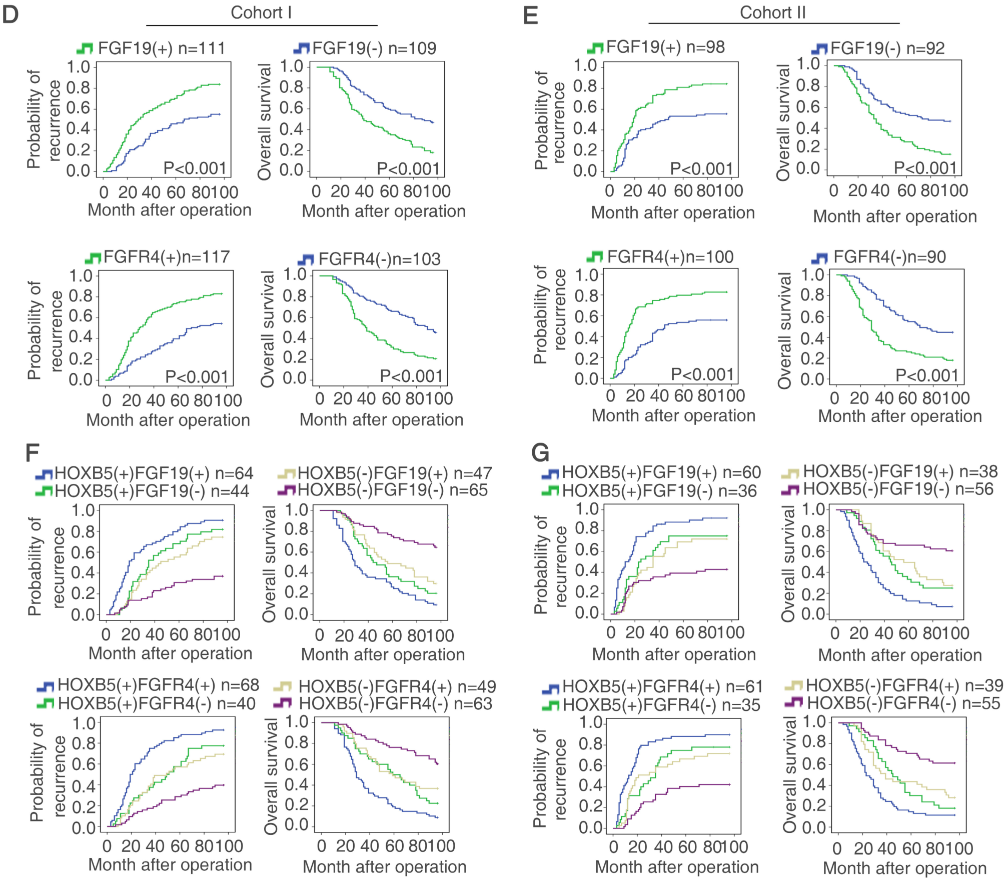

Figure S4. HOXB5 expression is positively correlated with FGF19 and FGFR4 expression in human HCC tissues.

(A) Representative IHC staining images showed FGF19, FGFR4 and HOXB5 expression in human HCC tissues.

(B-C) The correlation analysis of the expression of HOXB5 and FGF19 or HOXB5 and FGFR4 in human HCC tissues in cohort I (B) and cohort II (C).

(D-E) Overall survival time and probability of recurrence in HCC patients with positive or negative expression of FGF19 or FGFR4 in cohort I (D) and cohort II (E) were shown.

(F-G) Kaplan-Meier analyzed recurrence and overall survival time of patients with co-expression of FGF19/HOXB5 or FGFR4/HOXB5 in cohort I (F) and cohort II (G).

FigureS5.

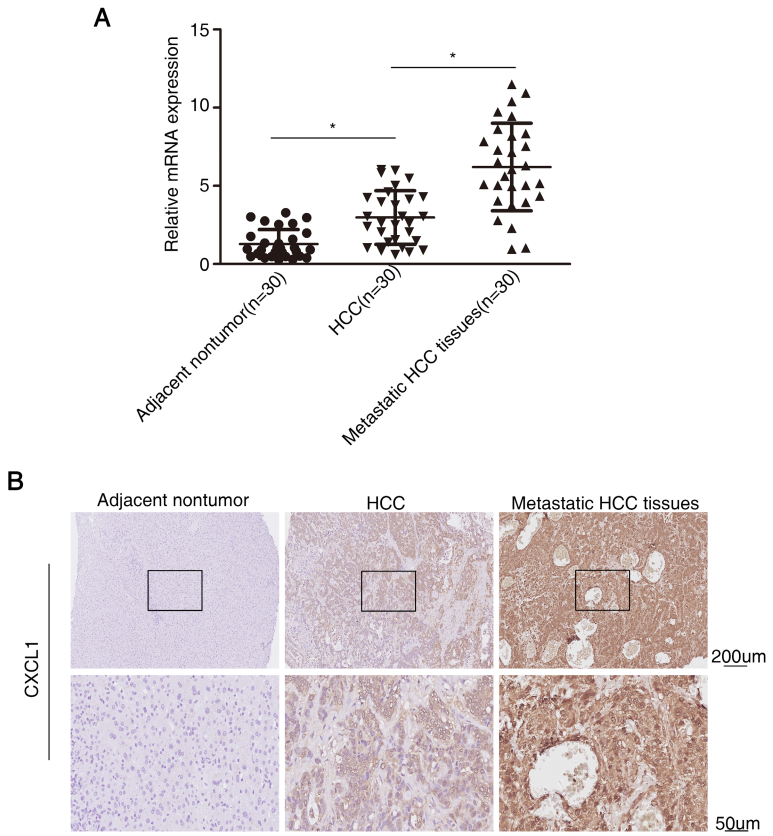

FigureS5: CXCL1 is upregulated in metastatic HCC tissues.

RT-qPCR (A) and IHC staining (B) showed CXCL1 expression in adjacent nontumor, HCC and Metastatic HCC tissues.

FigureS6

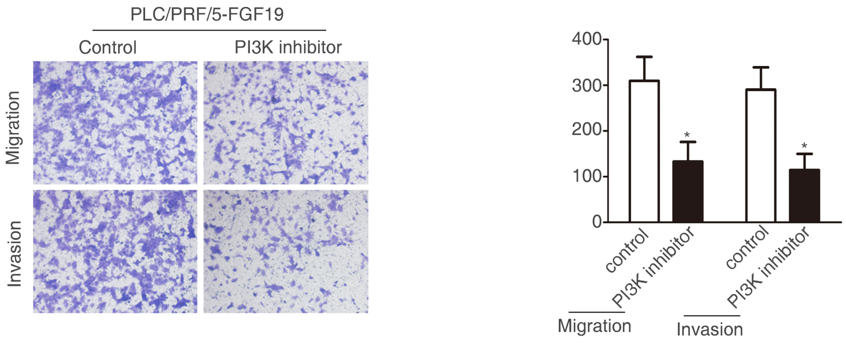

FigureS6: Transwell was used to demonstrate that PI3K inhibitor treatment inhibited the migratory and invasive abilities of PLC/PRF/5-FGF19 cells.

Table S1. Correlation between HOXB5 expression and clinicopathological characteristics of HCCs in two independent cohorts of human HCC tissues

		Cohort I			Cohort II		
		Tumor HOXB5 expression			Tumor HOXB5 expression		
Clinicopathological variables		Negative (n=112)	Positive (n=108)	*P* Value	Negative (n=94)	Positive (n=96)	*P* Value
Age		52.76(11.864)	51.01(12.666)	0.389	51.20(8.606)	50.65(7.654)	0.355
Sex	female	25	15	0.118	15	16	1.000
	male	87	93		79	80	
Serum AFP	≤20ng/ml	31	18	0.054	21	22	1.000
	>20ng/ml	81	90		73	74	
Virus infection	HBV	82	76	0.379	69	72	0.797
	HCV	12	13		11	8	
	HBV+HCV	3	8		3	5	
	none	15	11		11	11	
Cirrrhosis	absent	24	35	0.070	17	25	0.222
	present	88	73		77	71	
Child-pugh score	Class A	85	80	0.758	79	79	0.847
	Class B	27	28		15	17	
Tumor number	single	65	56	0.416	66	48	0.005
	multiple	47	52		28	48	
Maximal tumor size	≤5cm	53	30	0.003	59	48	0.081
	>5cm	59	78		35	48	
Tumor encapsulation	absent	39	60	0.003	17	48	<0.001
	present	73	48		77	48	
Microvascular invasion	absent	77	44	<0.001	73	30	<0.001
	present	35	64		21	66	
Tumor differentiation	I-II	97	74	0.002	80	53	<0.001
	III-Ⅳ	15	34		14	43	
TNM stage	I-II	92	62	<0.001	84	53	<0.001
	III	20	46		10	43	

Table S2. Univariate and multivariate analysis of factors associated with survival and recurrence in two independent cohorts of human HCC

	Time To Recurrence		Overall Survival	
Clinical Variables	HR(95% CI)	P value	HR(95% CI)	P value
**Cohort I(n=220)**				
**Univariate analysis**				
Age (≤50 versus > 50)	0.988(0.976-1.001)	0.077	0.987(0.974-1.000)	0.053
Sex (female versus male)	1.224(0.817-1.834)	0.327	1.261(0.840-1.892)	0.263
Serum AFP (≤20 versus >20 ng/ml)	0.705(0.472-1.051)	0.086	0.680(0.451-1.026)	0.066
HBV infection (no versus yes)	0.615(0.267-1.416)	0.254	0.655(0.284-1.506)	0.319
Cirrhosis (absent versus present)	1.054(0.739-1.504)	0.771	1.038(0.725-1.488)	0.838
Child-pugh score (A versus B)	1.201(0.826-1.745)	0.337	1.259(0.859-1.847)	0.238
Tumor number (single versus multiple)	0.491(0.357-0.675)	<0.001	0.460(0.333-0.637)	<0.001
Maximal tumor size (≤5cm versus >5)	0.661(0.472-0.925)	0.016	0.654(0.463-0.922)	0.015
tumor encapsulation (present versus absent)	2.627(1.902-3.626)	<0.001	2.728(1.966-3.784)	<0.001
Microvascular invasion (absent versus present)	0.386(0.279-0.533)	<0.001	0.367(0.264-0.509)	<0.001
Tumor differentiation (I-II versus III-IV)	0.513(0.356-0.740)	<0.001	0.481(0.333-0.695)	<0.001
TNM stage (I-II versus III)	0.089(0.060-0.132)	<0.001	0.082(0.055-0.123)	<0.001
HOXB5 expression (negative versus positive)	0.363(0.261-0.505)	<0.001	0.357(0.255-0.500)	<0.001
**Multivariate analysis**				
Tumor number (single versus multiple)	0.905(0.556-1.473)	0.688	0.826(0.509-1.341)	0.440
Maximal tumor size (≤5cm versus >5)	0.773(0.524-1.141)	0.195	0.792(0.532-1.178)	0.249
Tumor encapsulation (present versus absent)	1.437(0.921-2.241)	0.110	1.354(0.864-2.120)	0.186
Microvascular invasion (absent versus present)	0.742(0.477-1.152)	0.184	0.651(0.419-1.010)	0.055
Tumor differentiation (I-II versus III-IV)	1.283(0.853-1.929)	0.231	1.193(0.794-1.792)	0.396
TNM stage (I-II versus III)	0.112(0.065-0.194)	<0.001	0.110(0.063-0.191)	<0.001
HOXB5 expression (negative versus positive)	0.484(0.337-0.695)	<0.001	0.495(0.343-0.715)	<0.001
**Cohort II(n=190)**				
**Univariate analysis**				

Age (≤50 versus > 50)	1.002(0.981-1.023)	0.859	0.998(0.977-1.019)	0.854
Sex (female versus male)	1.317(0.851-2.039)	0.217	1.250(0.802-1.949)	0.324
Serum AFP (≤20 versus >20 ng/ml)	1.096(0.738-1.626)	0.650	1.143(0.770-1.696)	0.508
HBV infection (no versus yes)	1.170(0.425-3.222)	0.761	0.995(0.361-2.739)	0.992
Cirrhosis (absent versus present)	1.029(0.684-1.547)	0.891	1.080(0.721-1.617)	0.710
Child-pugh score (A versus B)	0.705(0.459-1.083)	0.111	0.683(0.444-1.050)	0.083
Tumor number (single versus multiple)	0.442(0.313-0.625)	<0.001	0.442(0.313-0.625)	<0.001
Maximal tumor size (≤5cm versus >5)	0.544(0.386-0.766)	<0.001	0.550(0.390-0.776)	0.001
Tumor encapsulation (present versus absent)	1.987(1.401-2.819)	<0.001	1.982(1.397-2.811)	<0.001
Microvascular invasion (absent versus present)	0.387(0.273-0.548)	<0.001	0.389(0.274-0.552)	<0.001
Tumor differentiation (I-II versus III-IV)	0.229(0.159-0.330)	<0.001	0.251(0.175-0.360)	<0.001
TNM stage (I-II versus III)	0.141(0.094-0.211)	<0.001	0.136(0.091-0.203)	<0.001
HOXB5 expression (negative versus positive)	0.394(0.277-0.562)	<0.001	0.376(0.262-0.538)	<0.001
**Multivariate analysis**				
Tumor number (single versus multiple)	0.825(0.533-1.276)	0.387	0.838(0.543-1.292)	0.423
Maximal tumor size (≤5cm versus >5)	0.960(0.642-1.435)	0.841	0.945(0.633-1.412)	0.783
Tumor encapsulation (present versus absent)	0.962(0.630-1.469)	0.858	1.006 (0.660-1.531)	0.980
Microvascular invasion (absent versus present)	0.817(0.510-1.311)	0.403	0.887(0.548-1.435)	0.624
Tumor differentiation (I-II versus III-IV)	0.546(0.332-0.897)	0.017	0.664(0.404-1.093)	0.107
TNM stage (I-II versus III)	0.308(0.165-0.574)	<0.001	0.267 (0.143-0.498)	<0.001
HOXB5 expression (negative versus positive)	0.644(0.429-0.968)	0.034	0.616(0.405-0.936)	0.023

Table S3. List of genes differentially expressed in PLC/PRF/5-HOXB5 versus PLC/PRF/5-control cells using a human metastasis and Cytokines & Chemokines PCR array

gene	Fold change	Description
**Metastasis PCR array**		
FGFR4	6.15	Fibroblast growth factor receptor 4
CXCR2	4.93	Chemokine receptor 2
MYC	4.46	V-myc myelocytomatosis viral oncogene homolog
CXCR4	4.03	Chemokine receptor 4
MMP10	3.84	Matrix metallopeptidase 10
MET	3.46	Met proto-oncogene
MTA1	3.31	Metastasis associated 1
CHD4	3.17	Chromodomain helicase DNA binding protein 4
MCAM	3.01	Melanoma cell adhesion molecule
COL4A2	2.72	Collagen, type IV, alpha 2
FN1	2.54	Fibronectin 1
FLT4	2.31	Fms-related tyrosine kinase 4
MMP7	2.14	Matrix metallopeptidase 7
CD44	2.06	CD44 molecule
CDH6	1.95	Cadherin 6, type 2, K-cadherin
VEGFA	1.83	Vascular endothelial growth factor A
CDH11	1.74	Cadherin 11, type 2, OB-cadherin
ETV4	1.65	Ets variant 4
HGF	1.65	Hepatocyte growth factor
IGF1	1.57	Insulin-like growth factor 1
IL1B	1.55	Interleukin 1, beta
MMP13	1.48	Matrix metallopeptidase 13
CCL7	1.45	Chemokine (C-C motif) ligand 7
IL18	1.45	Interleukin 18
CTBP1	1.41	C-terminal binding protein 1
DENR	1.37	Density-regulated protein
CXCL12	1.36	Chemokine ligand 12
CTSK	1.33	Cathepsin K
SYK	1.25	Spleen tyrosine kinase
TGFB1	1.25	Transforming growth factor, beta 1
SRC	1.24	V-src sarcoma viral oncogene homolog
MYCL1	1.21	V-myc myelocytomatosis viral oncogene homolog 1

MMP9	1.20	Matrix metallopeptidase 9
MMP2	1.15	Matrix metallopeptidase 2
CTSL1	1.13	Cathepsin L1
EPHB2	1.13	EPH receptor B2
ITGA7	1.12	Integrin, alpha 7
KRAS	1.10	V-Ki-ras2 Kirsten rat sarcoma viral oncogene homolog
MMP3	1.08	Matrix metallopeptidase 3
FAT1	1.07	FAT tumor suppressor homolog 1
METAP2	1.05	Methionyl aminopeptidase 2
MGAT5	1.05	Mannosyl (alpha-1,6-)-glycoprotein beta-1,6-N-acetyl-glucosaminyltransferase
SMAD2	1.03	SMAD family member 2
TCF20	1.02	Transcription factor 20
EWSR1	1.01	Ewing sarcoma breakpoint region 1
SMAD4	1.00	SMAD family member 4
MMP11	-1.01	Matrix metallopeptidase 11
ITGB3	-1.02	Integrin, beta 3
SERPINE1	-1.05	Serpin peptidase inhibitor, clade E, member 1
SET	-1.09	SET nuclear oncogene
TRPM1	-1.13	Transient receptor potential cation channel, subfamily M, member 1
RPSA	-1.15	Ribosomal protein SA
HRAS	-1.22	V-Ha-ras Harvey rat sarcoma viral oncogene homolog
PLAUR	-1.28	Plasminogen activator, urokinase receptor
TSHR	-1.28	Thyroid stimulating hormone receptor
PNN	-1.31	Pinin, desmosome associated protein
PTEN	-1.35	Phosphatase and tensin homolog
APC	-1.38	Adenomatous polyposis coli
CD82	-1.41	CD82 molecule
CST7	-1.43	Cystatin F
BRMS1	-1.46	Breast cancer metastasis suppressor 1
HPSE	-1.54	Heparanase
HTATIP2	-1.58	HIV-1 Tat interactive protein 2
TP53	-1.62	Tumor protein p53
RB1	-1.67	Retinoblastoma 1
RORB	-1.68	RAR-related orphan receptor B
CDKN2A	-1.75	Cyclin-dependent kinase inhibitor 2A
CTNNA1	-1.82	Catenin (cadherin-associated protein), alpha 1

MDM2	-1.88	Mdm2 p53 binding protein homolog
NF2	-1.94	Neurofibromin 2
NME1	-1.95	Non-metastatic cells 1
NR4A3	-1.97	Nuclear receptor subfamily 4, group A, member 3
NME4	-2.03	Non-metastatic cells 4
TNFSF10	-2.08	Tumor necrosis factor (ligand) superfamily, member 10
TIMP4	-2.14	TIMP metallopeptidase inhibitor 4
SSTR2	-2.19	Somatostatin receptor 2
FXYD5	-2.29	FXYD domain containing ion transport regulator 5
KISS1R	-2.53	KISS1 receptor
GNRH1	-2.68	Gonadotropin-releasing hormone 1
KISS1	-2.74	KiSS-1 metastasis-suppressor
TIMP2	-2.93	TIMP metallopeptidase inhibitor 2
MTSS1	-3.16	Metastasis suppressor 1
TIMP3	-3.38	TIMP metallopeptidase inhibitor 3
CDH1	-3.67	Cadherin 1, type 1, E-cadherin
**Cytokines & Chemokines PCR array**		
CXCL1	5.22	Chemokine (C-X-C motif) ligand 1
CSF1	4.36	Colony stimulating factor 1 (macrophage)
CCL2	4.03	Chemokine (C-C motif) ligand 2
CCL5	3.89	Chemokine (C-C motif) ligand 5
CCL20	3.84	Chemokine (C-C motif) ligand 20
CXCL2	3.76	Chemokine (C-X-C motif) ligand 2
BMP2	3.62	Bone morphogenetic protein 2
CCL24	3.59	Chemokine (C-C motif) ligand 24
CXCL11	3.50	Chemokine (C-X-C motif) ligand 11
CXCL5	3.32	Chemokine (C-X-C motif) ligand
SPP1	3.06	Secreted phosphoprotein 1
IL22	2.83	Interleukin 22
IL6	2.59	Interleukin 6
CCL3	2.42	Chemokine (C-C motif) ligand 3
BMP4	2.25	Bone morphogenetic protein 4
CXCL13	2.03	Chemokine (C-X-C motif) ligand 13
IL10	2.02	Interleukin 10
IL8	1.82	Interleukin 8
CCL1	1.77	Chemokine (C-C motif) ligand 1
CCL18	1.72	Chemokine (C-C motif) ligand 18
CCL17	1.64	Chemokine (C-C motif) ligand 17

MIF	1.55	Macrophage migration inhibitory factor
IL1B	1.52	Interleukin 1, beta
IL4	1.46	Interleukin 4
CXCL10	1.41	Chemokine (C-X-C motif) ligand 10
CX3CL1	1.37	Chemokine (C-X3-C motif) ligand 1
IL11	1.33	Interleukin 11
IL17A	1.31	Interleukin 17A
VEGFA	1.28	Vascular endothelial growth factor A
BMP6	1.26	Bone morphogenetic protein 6
CCL22	1.24	Chemokine (C-C motif) ligand 22
CCL7	1.24	Chemokine (C-C motif) ligand 7
CCL11	1.22	Chemokine (C-C motif) ligand 11
BMP7	1.20	Bone morphogenetic protein 7
CCL21	1.18	Chemokine (C-C motif) ligand 21
IL17F	1.16	Interleukin 17F
IL1A	1.12	Interleukin 1, alpha
IL5	1.12	Interleukin 5
IL9	1.11	Interleukin 9
TNF	1.09	Tumor necrosis factor
LTB	1.07	Lymphotoxin beta
CXCL12	1.07	Chemokine (C-X-C motif) ligand 12
CCL19	1.04	Chemokine (C-C motif) ligand 19
CCL8	1.03	Chemokine (C-C motif) ligand 8
CSF2	1.02	Colony stimulating factor 2
IL16	1.01	Interleukin 16
C5	1.00	Complement component 5
CNTF	-1.01	Ciliary neurotrophic factor
CSF3	-1.02	Colony stimulating factor 3
CCL13	-1.02	Chemokine (C-C motif) ligand 13
CD40LG	-1.04	CD40 ligand
ADIPOQ	-1.07	Adiponectin, C1Q and collagen domain containing
GPI	-1.09	Glucose-6-phosphate isomerase
CXCL16	-1.11	Chemokine (C-X-C motif) ligand 16
LTA	-1.17	Lymphotoxin alpha
MSTN	-1.22	Myostatin
PPBP	-1.23	Pro-platelet basic protein
TNFSF13B	-1.28	Tumor necrosis factor (ligand) superfamily, member 13b
XCL1	-1.37	Chemokine (C motif) ligand

THPO	-1.39	Thrombopoietin
LIF	-1.43	Leukemia inhibitory factor
IL3	-1.48	Interleukin 3
FASLG	-1.52	Fas ligand
IFNG	-1.56	Interferon, gamma
IFNA2	-1.59	Interferon, alpha 2
IL13	-1.65	Interleukin 13
IL1RN	-1.69	Interleukin 1 receptor antagonis
IL23A	-1.72	Interleukin 23, alpha subunit p19
NODAL	-1.72	Nodal homolog
TNFRSF11B	-1.84	Tumor necrosis factor receptor superfamily, member 11b
OSM	-1.87	Oncostatin M
TNFSF10	-1.96	Tumor necrosis factor (ligand) superfamily, member 1
TNFSF11	-2.01	Tumor necrosis factor (ligand) superfamily, member 11
IL12A	-2.03	Interleukin 12A
IL18	-2.11	Interleukin 18
IL12B	-2.28	Interleukin 12B
IL15	-2.42	Interleukin 15
IL7	-2.76	Interleukin 7
TGFB2	-2.82	Transforming growth factor, beta 2
IL21	-2.94	Interleukin 21
IL24	-3.03	Interleukin 24
IL2	-3.09	Interleukin 2
IL27	-3.25	Interleukin 27

Table S4. List of genes differentially expressed in MHCC97H-shHOXB5 versus MHCC97H-shcontrol cells using a human metastasis and Cytokines & Chemokines PCR array

gene	Fold change	Description
**Metastasis PCR array**		
FGFR4	-5.64	Fibroblast growth factor receptor 4
CHD4	-4.77	Chromodomain helicase DNA binding protein 4
MET	-4.28	Met proto-oncogene
CXCR2	-4.01	Chemokine receptor 2
MMP3	-3.91	Matrix metallopeptidase 3
VEGFA	-3.80	Vascular endothelial growth factor A
ETV4	-3.73	Ets variant 4
MYC	-3.51	V-myc myelocytomatosis viral oncogene homolog
MTA1	-3.38	Metastasis associated 1
HGF	-3.02	Hepatocyte growth factor
CCL7	-2.81	Chemokine (C-C motif) ligand 7
IL1B	-2.54	Interleukin 1, beta
TGFB1	-2.38	Transforming growth factor, beta 1
CXCL12	-2.29	Chemokine ligand 12
MMP9	-2.13	Matrix metallopeptidase 9
IL18	-2.06	Interleukin 18
CXCR4	-1.97	Chemokine receptor 4
MCAM	-1.97	Melanoma cell adhesion molecule
FLT4	-1.91	Fms-related tyrosine kinase 4
FN1	-1.82	Fibronectin 1
CD44	-1.81	CD44 molecule
MMP7	-1.75	Matrix metallopeptidase 7
COL4A2	-1.53	Collagen, type IV, alpha 2
MMP13	-1.49	Matrix metallopeptidase 13
CDH6	-1.46	Cadherin 6, type 2, K-cadherin
MMP2	-1.46	Matrix metallopeptidase 2
SYK	-1.42	Spleen tyrosine kinase
MYCL1	-1.33	V-myc myelocytomatosis viral oncogene homolog 1
MMP10	-1.32	Matrix metallopeptidase 10
MMP11	-1.31	Matrix metallopeptidase 11
ITGA7	-1.25	Integrin, alpha 7
CTSK	-1.24	Cathepsin K

DENR	-1.24	Density-regulated protein
SRC	-1.22	V-src sarcoma viral oncogene homolog
IGF1	-1.18	Insulin-like growth factor 1
CDH11	-1.16	Cadherin 11, type 2, OB-cadherin
CTBP1	-1.16	C-terminal binding protein 1
SET	-1.14	SET nuclear oncogene
TSHR	-1.12	Thyroid stimulating hormone receptor
APC	-1.11	Adenomatous polyposis coli
HRAS	-1.08	V-Ha-ras Harvey rat sarcoma viral oncogene homolog
MDM2	-1.07	Mdm2 p53 binding protein homolog
TRPM1	-1.07	Transient receptor potential cation channel, subfamily M, member 1
CD82	-1.05	CD82 molecule
ITGB3	-1.03	Integrin, beta 3
PTEN	-1.01	Phosphatase and tensin homolog
SMAD2	1.00	SMAD family member 2
SMAD4	1.04	SMAD family member 4
PLAUR	1.06	Plasminogen activator, urokinase receptor
TCF20	1.09	Transcription factor 20
FAT1	1.11	FAT tumor suppressor homolog 1
CTSL1	1.14	Cathepsin L1
MGAT5	1.16	Mannosyl (alpha-1,6-)-glycoprotein beta-1,6-N-acetyl-glucosaminyltransferase
EWSR1	1.16	Ewing sarcoma breakpoint region 1
SERPINE1	1.18	Serpin peptidase inhibitor, clade E, member 1
PNN	1.22	Pinin, desmosome associated protein
METAP2	1.23	Methionyl aminopeptidase 2
EPHB2	1.28	EPH receptor B2
RPSA	1.30	Ribosomal protein SA
KRAS	1.36	V-Ki-ras2 Kirsten rat sarcoma viral oncogene homolog
TP53	1.38	Tumor protein p53
CDKN2A	1.44	Cyclin-dependent kinase inhibitor 2A
HPSE	1.45	Heparanase
RORB	1.51	RAR-related orphan receptor B
SSTR2	1.51	Somatostatin receptor 2
NME4	1.54	Non-metastatic cells 4
CST7	1.63	Cystatin F
RB1	1.68	Retinoblastoma 1

BRMS1	1.72	Breast cancer metastasis suppressor 1
CTNNA1	1.74	Catenin (cadherin-associated protein), alpha 1
HTATIP2	1.81	HIV-1 Tat interactive protein 2
MTSS1	1.84	Metastasis suppressor 1
NF2	1.88	Neurofibromin 2
TNFSF10	1.95	Tumor necrosis factor (ligand) superfamily, member 10
KISS1R	2.03	KISS1 receptor
TIMP4	2.05	TIMP metallopeptidase inhibitor 4
NR4A3	2.29	Nuclear receptor subfamily 4, group A, member 3
FXYD5	2.34	FXYD domain containing ion transport regulator 5
NME1	2.39	Non-metastatic cells 1
CDH1	2.69	Cadherin 1, type 1, E-cadherin
GNRH1	2.77	Gonadotropin-releasing hormone 1
KISS1	2.91	KiSS-1 metastasis-suppressor
TIMP2	3.15	TIMP metallopeptidase inhibitor 2
TIMP3	3.58	TIMP metallopeptidase inhibitor 3
**Cytokines & Chemokines PCR array**		
CXCL1	-5.53	Chemokine (C-X-C motif) ligand 1
CXCL2	-4.41	Chemokine (C-X-C motif) ligand 2
CXCL5	-4.18	Chemokine (C-X-C motif) ligand
IL8	-3.82	Interleukin 8
CCL2	-3.74	Chemokine (C-C motif) ligand 2
SPP1	-3.52	Secreted phosphoprotein 1
VEGFA	-3.41	Vascular endothelial growth factor A
CSF1	-2.85	Colony stimulating factor 1 (macrophage)
CCL18	-2.64	Chemokine (C-C motif) ligand 18
BMP7	-2.61	Bone morphogenetic protein 7
CCL1	-2.51	Chemokine (C-C motif) ligand 1
IL1B	-2.41	Interleukin 1, beta
CXCL16	-2.36	Chemokine (C-X-C motif) ligand 16
CCL17	-2.15	Chemokine (C-C motif) ligand 17
CXCL10	-2.07	Chemokine (C-X-C motif) ligand 10
IL18	-2.04	Interleukin 18
IL22	-1.94	Interleukin 22
CCL3	-1.91	Chemokine (C-C motif) ligand 3
CXCL13	-1.85	Chemokine (C-X-C motif) ligand 13
CXCL11	-1.81	Chemokine (C-X-C motif) ligand 11
IL1A	-1.74	Interleukin 1, alpha

IL6	-1.72	Interleukin 6
IL10	-1.67	Interleukin 10
MIF	-1.65	Macrophage migration inhibitory factor
CCL7	-1.62	Chemokine (C-C motif) ligand 7
IL4	-1.57	Interleukin 4
IL17A	-1.52	Interleukin 17A
CCL13	-1.44	Chemokine (C-C motif) ligand 13
CCL5	-1.41	Chemokine (C-C motif) ligand 5
BMP2	-1.40	Bone morphogenetic protein 2
CXCL12	-1.36	Chemokine (C-X-C motif) ligand 12
CCL22	-1.31	Chemokine (C-C motif) ligand 22
CCL20	-1.28	Chemokine (C-C motif) ligand 20
IL11	-1.24	Interleukin 11
BMP4	-1.21	Bone morphogenetic protein 4
CD40LG	-1.20	CD40 ligand
CCL11	-1.18	Chemokine (C-C motif) ligand 11
CSF3	-1.18	Colony stimulating factor 3
XCL1	-1.16	Chemokine (C motif) ligand
LIF	-1.13	Leukemia inhibitory factor
IL9	-1.10	Interleukin 9
CSF2	-1.08	Colony stimulating factor 2
CD40LG	-1.07	CD40 ligand
GPI	-1.05	Glucose-6-phosphate isomerase
CNTF	-1.03	Ciliary neurotrophic factor
LTA	-1.01	Lymphotoxin alpha
C5	1.00	Complement component 5
IL5	1.05	Interleukin 5
CCL21	1.07	Chemokine (C-C motif) ligand 21
LTB	1.09	Lymphotoxin beta
CCL24	1.12	Chemokine (C-C motif) ligand 24
IL16	1.14	Interleukin 16
IL17F	1.16	Interleukin 17F
BMP6	1.21	Bone morphogenetic protein 6
CCL8	1.24	Chemokine (C-C motif) ligand 8
CX3CL1	1.26	Chemokine (C-X3-C motif) ligand 1
PPBP	1.29	Pro-platelet basic protein
TNF	1.31	Tumor necrosis factor
CCL19	1.35	Chemokine (C-C motif) ligand 19
IL3	1.42	Interleukin 3

MSTN	1.43	Myostatin
ADIPOQ	1.48	Adiponectin, C1Q and collagen domain containing
IL12A	1.55	Interleukin 12A
IL1RN	1.58	Interleukin 1 receptor antagonis
IFNG	1.63	Interferon, gamma
IL23A	1.68	Interleukin 23, alpha subunit p19
TNFSF10	1.72	Tumor necrosis factor (ligand) superfamily, member 1
FASLG	1.77	Fas ligand
OSM	1.82	Oncostatin M
TGFB2	1.86	Transforming growth factor, beta 2
IFNA2	1.93	Interferon, alpha 2
IL12B	1.97	Interleukin 12B
IL13	2.02	Interleukin 13
TNFSF13B	2.02	Tumor necrosis factor (ligand) superfamily, member 13b
TNFRSF11B	2.09	Tumor necrosis factor receptor superfamily, member 11b
TNFSF11	2.15	Tumor necrosis factor (ligand) superfamily, member 11
IL2	2.31	Interleukin 2
THPO	2.41	Thrombopoietin
IL15	2.49	Interleukin 15
NODAL	2.58	Nodal homolog
IL24	2.83	Interleukin 24
IL27	2.95	Interleukin 27
IL7	3.02	Interleukin 7
IL21	3.28	Interleukin 21

Table S5. Correlation between CXCL1 expression and clinicopathological characteristics of HCCs in two independent cohorts of human HCC tissues

Clinicopathological variables		Cohort I			Cohort II		
		Tumor CXCL1 expression			Tumor CXCL1 expression		
		Negative (n=87)	Positive (n=133)	*P* Value	Negative (n=72)	Positive (n=118)	*P* Value
Age		53.89(11.801)	50.60(12.436)	0.385	51.75(7.925)	50.42(8.232)	0.608
Sex	female	17	23	0.722	11	20	0.841
	male	70	110		61	98	
Serum AFP	≤20ng/ml	21	28	0.621	16	27	1.000
	>20ng/ml	66	105		56	91	
Virus infection	HBV	68	90	0.252	58	83	0.215
	HCV	7	18		4	15	
	HBV+HCV	5	6		4	4	
	none	7	19		6	16	
Cirrhosis	absent	26	33	0.439	13	29	0.368
	present	61	100		59	89	
Child-pugh score	Class A	67	98	0.635	59	99	0.842
	Class B	20	35		13	19	
Tumor number	single	55	66	0.053	51	63	0.022
	multiple	32	67		21	55	
Maximal tumor size	≤5cm	37	46	0.257	49	58	0.016
	>5cm	50	87		23	60	
Tumor encapsulation	absent	29	70	0.006	15	50	0.003
	present	58	63		57	68	
Microvascular invasion	absent	63	58	<0.001	55	48	<0.001
	present	24	75		17	70	
Tumor differentiation	I-II	75	96	0.020	62	71	<0.001
	III-Ⅳ	12	37		10	47	
TNM stage	I-II	73	81	<0.001	63	74	<0.001
	III	14	52		9	44	

Table S6. Correlation between CD11b expression and clinicopathological characteristics of HCCs in two independent cohorts of human HCC tissues

Clinicopathological variables		Cohort I Tumor CD11b expression Negative (n=159)	Cohort I Tumor CD11b expression Positive (n=61)	Cohort I *P* Value	Cohort II Tumor CD11b expression Negative (n=137)	Cohort II Tumor CD11b expression Positive (n=53)	Cohort II *P* Value
Age		51.86(12.232)	52.02(12.461)	0.672	50.55(8.413)	51.87(7.303)	0.446
Sex	female	30	10	0.845	25	6	0.282
	male	129	51		112	47	
Serum AFP	≤20ng/ml	39	10	0.211	31	12	1.000
	>20ng/ml	120	51		106	41	
Virus infection	HBV	117	41	0.677	102	39	0.852
	HCV	16	9		13	6	
	HBV+HCV	7	4		5	3	
	none	19	7		17	5	
Cirrrhosis	absent	39	20	0.236	29	13	0.697
	present	120	41		108	40	
Child-pugh score	Class A	119	46	1.000	114	44	1.000
	Class B	40	15		23	9	
Tumor number	single	94	27	0.051	87	27	0.138
	multiple	65	34		50	26	
Maximal tumor size	≤5cm	66	17	0.065	90	17	<0.001
	>5cm	93	44		47	36	
Tumor encapsulation	absent	60	39	0.001	39	26	0.010
	present	99	22		98	27	
Microvascular invasion	absent	99	22	0.001	88	15	<0.001
	present	60	39		49	38	
Tumor differentiation	I-II	134	37	<0.001	110	23	<0.001
	III-Ⅳ	25	24		27	30	
TNM stage	I-II	125	29	<0.001*	113	24	<0.001*
	III	34	32		24	29	

Table S7. Correlation between FGF19 expression and clinicopathological characteristics of HCCs in two independent cohorts of human HCC tissues

		Cohort I			Cohort II		
		Tumor FGF19 expression			Tumor FGF19 expression		
Clinicopathological variables		Negative (n=109)	Positive (n=111)	*P* Value	Negative (n=92)	Positive (n=98)	*P* Value
Age		54.03(12.400)	49.81(11.823)	0.617	50.52(8.180)	51.30(8.091)	0.843
Sex	female	19	21	0.862	15	16	1.000
	male	90	90		77	82	
Serum AFP	≤20ng/ml	26	23	0.629	19	24	0.604
	>20ng/ml	83	88		73	74	
Virus infection	HBV	82	76	0.053	69	72	0.951
	HCV	6	19		8	11	
	HBV+HCV	6	5		4	4	
	none	15	11		11	11	
Cirrrhosis	absent	26	33	0.363	15	27	0.080
	present	83	78		77	71	
Child-pugh score	Class A	85	80	0.352	77	81	1.000
	Class B	24	31		15	17	
Tumor number	single	63	58	0.420	58	56	0.460
	multiple	46	53		34	42	
Maximal tumor size	≤5cm	42	41	0.889	63	44	0.001
	>5cm	67	70		29	54	
Tumor encapsulation	absent	35	64	<0.001	21	44	0.002
	present	74	47		71	54	
Microvascular invasion	absent	74	47	<0.001	65	38	<0.001
	present	35	64		27	60	
Tumor differentiation	I-II	94	77	0.003	77	56	<0.001
	III-Ⅳ	15	34		15	42	
TNM stage	I-II	88	66	0.001*	80	57	<0.001*
	III	21	45		12	41	

Table S8. Correlation between FGFR4 expression and clinicopathological characteristics of HCCs in two independent cohorts of human HCC tissues

		Cohort I			Cohort II		
		Tumor FGFR4 expression			Tumor FGFR4 expression		
Clinicopathological variables		Negative (n=103)	Positive (n=117)	*P* Value	Negative (n=90)	Positive (n=100)	*P* Value
Age		53.21(12.238)	50.74(12.230)	0.963	51.20(8.672)	50.67(7.629)	0.266
Sex	female	18	22	0.862	11	20	0.171
	male	85	95		79	80	
Serum AFP	≤20ng/ml	21	28	0.627	22	21	0.606
	>20ng/ml	82	89		68	79	
Virus infection	HBV	77	81	0.477	69	72	0.183
	HCV	10	15		6	13	
	HBV+HCV	3	8		6	2	
	none	13	13		9	13	
Cirrhosis	absent	28	31	1.000	22	20	0.488
	present	75	86		68	80	
Child-pugh score	Class A	75	90	0.534	75	83	1.000
	Class B	28	27		15	17	
Tumor number	single	63	58	0.103	56	58	0.657
	multiple	40	59		34	42	
Maximal tumor size	≤5cm	43	40	0.267	64	43	<0.001
	>5cm	60	77		26	57	
Tumor encapsulation	absent	35	64	0.003	22	43	0.009
	present	68	53		68	57	
Microvascular invasion	absent	66	55	0.014	66	37	<0.001
	present	37	62		24	63	
Tumor differentiation	I-II	87	84	0.034	78	55	<0.001
	III-Ⅳ	16	33		12	45	
TNM stage	I-II	87	67	<0.001*	80	57	<0.001*
	III	16	50		10	43	

Table S9. Primer sequences used in the study

Primer name	Primer sequences	Enzyme
Primers for real-time PCR:		
HOXB5 sense:	5'- CGGCTACAATTACAATGGGATG-3'	
HOXB5 antisense:	5'- GGCCTCGTCTATTTCGGTGA-3'	
FGFR4 sense:	5’- GAGGGGCCGCCTAGAGATT-3’	
FGFR4 antisense:	5’- CAGGACGATCATGGAGCCT-3’	
CXCL1 sense:	5’- AGAAGGTGTTGAGCGGGAAG-3’	
CXCL1 antisense:	5’- TGAGACGAGAAGGAGCATTGG-3’	
β-actin sense:	5’-CATGTACGTTGCTATCCAGGC -3’	
β-actin antisense:	5’-CTCCTTAATGTCACGCACGAT -3’	
Primers for FGFR4 promoter construct:		
(-1781/138bp) FGFR4 sense:	5’- TATAGGTACCGGAGACCATTCTTTCGCT -3’	KpnI
(-1335/+138bp) FGFR4 sense:	5’- TATAGGTACCTGGCCACAGTCTAGATGC -3’	KpnI
(-765/+138bp) FGFR4 sense:	5’- TATAGGTACCTCACAGTAGAGACGTCAT -3’	KpnI
(-186/+138) FGFR4 sense:	5’- TATAGGTACCACCTCTCTCCGGCTCGAG-3’	KpnI
Antisense:	5’- ATATACGCGTAGCACAGGGTCCTGCTCA -3’	MluI
Primers for FGFR4 promoter site-directed mutagenesis:		
HOXB5 binding site:		
binding site 6 mutation sense:	5’-CTTGCCTGCGTAACgcgtAGAAAGGAAGTTGC-3’	
binding site 6 mutation antisense:	5’-GCAACTTCCTTTCTacgcGTTACGCAGGCAAG-3’	
binding site 5 mutation sense:	5’-GACACTTGAACTCTgcgtGTGAGGTCTGAGGA-3’	
binding site 5 mutation antisense:	5’-TCCTCAGACCTCACacgcAGAGTTCAAGTGTC-3’	
binding site 4 mutation sense:	5’-CTACAGGCACCCGTagcgGTGCCCGGCCATTC-3’	
binding site 4 mutation antisense:	5’-GAATGGCCGGGCACcgctACGGGTGCCTGTAG-3’	
binding site 3 mutation sense:	5’-ACAGCTATTTACATgccgAACTGTTCAGTTAC-3’	
binding site 3 mutation antisense:	5’-GTAACTGAACAGTTcggcATGTAAATAGCTGT-3’	
binding site 2 mutation sense:	5’-AACAGGTCCTAAATgccgACAGCTAGTATTAC-3’	
binding site 2 mutation antisense:	5’-GTAATACTAGCTGTcggcATTTAGGACCTGTT-3’	
binding site 1 mutation sense:	5’-CAGGAGGCGTGGGTagctGCAATCTATGTATA-3’	
binding site 1 mutation antisense:	5’-TATACATAGATTGCagctACCCACGCCTCCTG-3’	
Primers used for ChIP in the FGFR4 promoter:		
distant region sense:	5’-GACGAGATCTCACTATGT-3’	
distant region antisense:	5’-TTGCATAACTGTAAGGCC-3’	
binding site 1 sense:	5’-CTTCCTGAAGGAAGTGAG-3’	
binding site 1 antisense:	5’-CACAAATCTGACTGTGTC-3’	
Primers for CXCL1 promoter construct:		
(-1975/+268bp) CXCL1 sense:	5’-TATACTCGAGCTGGAGTACATATACTAT-3’	XhoI
(-1482/+268bp) CXCL1 sense:	5’-TATACTCGAGCTCATAACCACCTTGGGG-3’	XhoI
(-1051/+268bp) CXCL1 sense:	5’-TATACTCGAGTGACTCTATGTGGGATCC-3’	XhoI
(-403/+268 bp) CXCL1 sense:	5’-TATACTCGAGAAGAGGTCGCGCCTTCTC-3’	XhoI
Antisense:	5’-ATATAAGCTTGAAGAGACTCACTGACTG-3’	HindIII
Primers for CXCL1 promoter site-directed mutagenesis:		
HOXB5 binding site:		

binding site 3 mutation sense:	5’-ACTAAGATTACTGTgcgaAGAGCACCAGTACC-3’	
binding site 3 mutation antisense:	5’-GGTACTGGTGCTCTtcgcACAGTAATCTTAGT-3’	
binding site 2 mutation sense:	5’-CCCTCTAAAAACCTcggtATGAATAAAAATAA -3’	
binding site 2 mutation antisense:	5’-TTATTTTTATTCATaccgAGGTTTTTAGAGGG -3’	
binding site 1 mutation sense:	5’-AGGAATGGTGAGCCtggaAGCCTGCTGGACCC -3’	
binding site 1 mutation antisense:	5’-GGGTCCAGCAGGCTtccaGGCTCACCATTCCT -3’	
Primers used for ChIP in the CXCL1 promoter:		
distant region sense:	5’- TGAGCATATATTACAACT -3’	
distant region antisense:	5’- TGTGACTAGCTCATGCAT -3’	
binding site 3 sense:	5’- GAAACAACATTCTAGCAC -3’	
binding site 3 antisense:	5’- CAGCAGTATGAGTCTTGA -3’	
binding site 2/1 sense:	5’- GGCAGAAGCCTCTGCTTT -3’	
binding site 2/1 antisense:	5’- ACTGGTTGTCACTCCGGA -3’	
Primers for HOXB5 promoter construct:		
(-1460/+150bp) HOXB5 sense:	5’- TATAGGTACCATCCTGGCTAACACGATG -3’	KpnI
(-962/+150bp) HOXB5 sense:	5’- TATAGGTACCCAGGTGACCTAGACAGCT -3’	KpnI
(-444/+150bp) HOXB5 sense:	5’- TATAGGTACCGATCCTGCACTGATCTGG -3’	KpnI
(-67/+150bp) HOXB5 sense:	5’- TATAGGTACCGGCTTCTGAATTGTCCTC-3’	KpnI
antisense:	5’-TATAACGCGTTATCTGGCCGATCAGGTT-3’	MluI
Primers for HOXB5 promoter site-directed mutagenesis:		
HIF1α binding site:		
binding site 1 mutation sense:	5’-GGTCAACAAAAGCgtacGATTCCCTAACGC -3’	
binding site 1 mutation antisense:	5’-GCGTTAGGGAATCgtacGCTTTTGTTGACC -3’	
binding site 2 mutation sense:	5’-TCTGTCACTCCCCggaCCTGGGCTAAGGA -3’	
binding site 2 mutation antisense:	5’-TCCTTAGCCCAGGtccGGGGAGTGACAGA -3’	
ATF2 binding site:		
binding site mutation sense:	5’-GCATGAATTACCTCTTactaTCATCAGCGAGAATTTA-3’	
binding site mutation antisense:	5’-TAAATTCTCGCTGATGAtagtAAGAGGTAATTCATGC-3’	
Primers used for ChIP in the HOXB5 promoter:		
distant region sense:	5’- AACTGCCTAGCTGCGAGT -3’	
distant region antisense:	5’- AGCGGCCTAGTTGCCGAA -3’	
binding site 1/2 sense:	5’- CAATTCGGAGACTTGCAA -3’	
binding site 1/2 antisense:	5’- CGAAGTACAGTGCATCGC -3’	

Table S10. Knockdown shRNA sequences used in this study

shRNA genes	Sequence
shHOXB5-1	CCGGGAAGAAGGACAACAAATTGAACTCGAGTTCAATTTGTTGTCCTTCTTCTTTTTG
shHOXB5-2	CCGGCGGCTACAATTACAATGGGATCTCGAGATCCCATTGTAATTGTAGCCGTTTTT
shHOXB5-3	CCGGGCTTCACATCAGCCATGATATCTCGAGATATCATGGCTGATGTGAAGCTTTTT
shHOXB5-mouse	CCGGGCTTCACATCAGCCACGATATCTCGAGATATCGTGGCTGATGTGAAGCTTTTTG
shFGFR4-human	CCGGCCCTCGAATAGGCACAGTTACCTCGAGGTAACTGTGCCTATTCGAGGGTTTTTG
shFGFR4-mouse	CCGGGCTGTCTCTGAAGAGTACCTTCTCGAGAAGGTACTCTTCAGAGACAGCTTTTT
shCXCL1-mouse	CCGGCAAACCGAAGTCATAGCCACACTCGAGTGTGGCTATGACTTCGGTTTGTTTTTG

Table S11. The binding sites of HIF1α and ATF2 in HOXB5 promoter

HOXB5 promoter (-444bp ~ +150bp)

AAGGCTCAACCCTGTGGCTGAGCCTTGGCCGGGTTTCTTTCTCTCCCGGCGACTGCAG

GGAGCCTGCAGGGAGCCAGCAGGGAGGCAGCCGCCCGCCGCCATGGCGGTCTCACGC

HIF1α binding site-2

CCGCGCTCCCGTCGGTCGCCGGGAGGTGGCGCGCTCTGCTCAGAGGCCAAAGCCAAC

CTTCTCTCTGTTTCTCCCCCTTCTCCTTTTTTCCCCCTCTCTCGGAGGCTCGCATTGAAT

CGGCCCTAACGATTCTCGGATCGTCATTATTTGTAACCATAGAGCATGAATTACCTCTTG

AGGTCATCAGCGAGAATTTACGACTGGTCAACAAAAGCACGTGATTCCCTAACGCCCC

ATF2 binding site **HIF1α binding site-1**

CCACCCCCCTTCCAACCCCCCCCCCATATTTGGCCGCATACATAGCAAAACGAAGTACA

GTGCATCGCTATAATTCATTAATACATCATAAATCGTGAAGCACAGGGTTATAACGACCA

Transcription starting region

CGATCCACAAATCAAGCCCTCCAAAATCACCCAAATGAGCTCGTACTTTGTAAACTCCT

TCTCGGGGCGTTATCCAAATGGCCCGGACTATCAGTTGCTAAATTATGGCAGTGGCAGC

TCTCTGA
